# Ultrafast dynamics in core-excited states probed by resonant Auger spectroscopy: pyrrole

**DOI:** 10.1039/d5sc09051b

**Published:** 2026-02-27

**Authors:** D. M. P. Holland, H. G. McGhee, M. Lamanec, D. Nachtigallová, A. Milosavljević, J. D. Bozek, E. Muchová, R. A. Ingle

**Affiliations:** a STFC, Daresbury Laboratory Daresbury Warrington Cheshire WA4 4AD UK; b Department of Chemistry 20 Gordon Street London WC1H 0AJ UK; c Institute of Organic Chemistry and Biochemistry, Czech Academy of Sciences Flemingovo Náměstí 542/2 16000 Prague Czech Republic; d IT4Innovations, VŠB-Technical University of Ostrava 17. Listopadu 2172/15 70800 Ostrava-Poruba Czech Republic; e Synchrotron SOLEIL L'Orme des Merisiers Gif-sur-Yvette 91192 France; f Department of Physical Chemistry, University of Chemistry and Technology in Prague Technická 5 Prague 166 28 Czech Republic muchovae@vscht.cz; g Department of Chemistry 20 Gordon Street London WC1H 0AJ UK r.ingle@ucl.ac.uk

## Abstract

We present high-resolution resonant Auger spectra of pyrrole at both the nitrogen and carbon edges. Through comparison to high-level quantum chemical calculations and a linear vibronic coupling model, we show how resonant Auger spectroscopy can capture the out-of-plane nuclear motions that occur during the few femtosecond core–hole relaxation processes. We also demonstrate how, in scenarios where there are near-degenerate electronic transitions in the X-ray absorption spectrum that cannot be resolved experimentally, resonant Auger can be used as a probe of the underlying electronic structure. Overall, resonant Auger is a technique capable of revealing detailed information about core-excited molecules that cannot be recovered through X-ray absorption or photoelectron spectroscopy alone.

## Abstract 2

High-resolution resonant Auger spectra of pyrrole at the carbon edge reveals information on ultrafast dynamics in the core-excited states.

## Graphical abstract

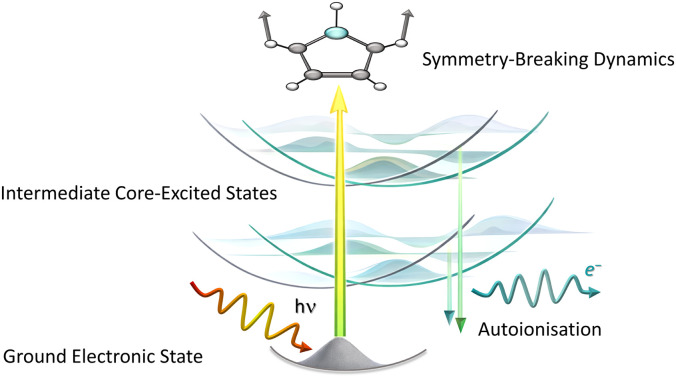

## Introduction

The chemical and physical properties of molecules are encoded in their electronic structure. Spectroscopic studies often aim to understand the electronic structure of a molecule in a ‘chemically intuitive’ framework, for example, by relating the peaks in the experimental spectrum to visualisations of molecular orbitals. However, even for medium-size molecules, many experimental spectra have a large number of overlapping features that make meaningful spectral decomposition challenging.

**Fig. 1:**
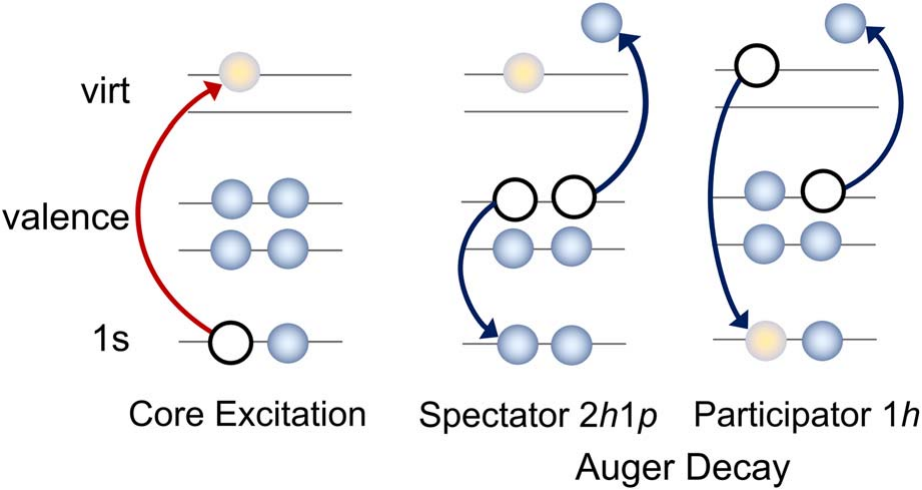
Sketch illustrating the resonant Auger process and two potential decay pathways that lead to different electronic configurations in the final state.

X-ray spectroscopies are element and site selective, which reduces the number of contributions to the final spectrum, and can simplify experimental interpretation as any observed peaks must be related to a particular element. Resonant X-ray methods, such as resonant inelastic X-ray scattering (RIXS) or resonant Auger electron spectroscopy (RAES), not only have enhanced selectivity in comparison to methods like X-ray absorption spectroscopy (XAS) or X-ray photoelectron spectroscopy (XPS), as well as improved spectral resolution, but also provide additional information on the ultrafast dynamics of core-excited states due to the nature of the probing process. Resonant X-ray techniques can be viewed as a multi-step process, where a core electron is initially excited to an unoccupied valence orbital. This intermediate core-excited state is highly unstable and so undergoes core–hole relaxation on a timescale that may be as short as a few femtoseconds and results in the ejection of an Auger electron (RAES, see Fig. 1) or photon (RIXS). In contrast to XAS or XPS, the final Auger electron spectrum is unaffected by the significant core–hole lifetime broadening associated with the core-excited state. If a sufficiently narrow bandwidth excitation pulse is used, resonant X-ray methods provide unique selectivity with respect to the electronic and vibrational states, and provide a clear view of the character, and the charge and nuclear dynamics, of the core excited state.

RAES has been used as a tool for the characterisation of molecular electronic structure for both ground state and photoexcited molecules,^1–3^ as well as for the measurement of electron delocalisation^4^ and charge injection dynamics^5^*via* core–hole-clock techniques. However, the main advantage of RAES – its sensitivity to electronic or vibrational states – is at the same time its curse. RAES does not allow for a straightforward and chemically intuitive interpretation, *e. g.* the measured signal does not correspond to a simple observable which can be related to a conceptually simple process (such as the change in the character of an electronically excited state as in XAS, or the change in the electronic structure due to chemical bonding as in XPS). The theoretical interpretation is also further complicated as the calculations need to take into account the outgoing Auger electron (which is not a square-integrable function) to estimate the Auger rates.

In this work, we report experimental Auger spectra of gas-phase pyrrole at the carbon and nitrogen K-edges, with accompanying electronic structure calculations of the X-ray absorption and resonant Auger observables. Together, these results provide insight into how the participator and spectator Auger signals can be used to unambiguously distinguish different electronic state contributions that cannot be determined from the absorption spectrum alone. Using state-of-the-art theoretical approaches, combined with molecular orbital visualisations, we provide a more ‘chemically intuitive’ framework for the interpretation of RAES signals useful especially for a broader audience. Moreover, after core-excitation of the equivalent carbon atoms, pyrrole inevitably undergoes symmetry-breaking dynamics. We demonstrate that even though the dynamics cannot be directly observed in XAS due to core–hole lifetime broadening, it clearly manifests in the resonant Auger spectra measured at different photon energies.

As well as being a common structural motif in chemistry, pyrrole is an excellent example of a molecule where core-excitation results in the population of structurally and spatially different orbitals (the lowest unoccupied molecular orbitals are of antibonding π* character, or σ* combined with Rydberg character) which is common to many heterocyclic systems. The photochemistry of these compounds is largely mediated by the character of the unoccupied orbitals and their typical vibrational motion (for example, ring-puckering vibrations) which can lead to σ bond breaking or ring opening. In this respect, pyrrole provides universal results applicable to many structurally similar systems.

## Methods

### Experimental methods

The photoelectron and Auger electron spectra were recorded with a Scienta R4000 hemispherical electron energy analyzer attached to the PLÉIADES beamline at the SOLEIL synchrotron radiation facility. Detailed descriptions of the beamline and endstation instrumentation have been reported previously^6–8^ so only details relevant to the present work will be reported here.

The monochromator exit slit was set to provide optical resolutions of ∼3–10 meV, 35 meV and 50 meV, respectively, for the valence, carbon and nitrogen photoelectron spectra, while an optical resolution of 10–120 meV was used for the resonantly excited Auger spectra. The Scienta analyser pass energies and slit widths were chosen to provide resolutions of ∼10 meV, and 10–25 meV for the valence and core shell photoelectron spectra, and 40–120 meV for the resonantly excited Auger spectra. Total ion yields, which are assumed to resemble the photoabsorption spectra, were measured at the C and N edges using optical resolutions of ∼23 and 38 meV, respectively. Photoelectron and Auger electron spectra were measured with the electric vector of the linearly polarised radiation lying either parallel or perpendicular to the acceptance axis of the electron spectrometer. So-called magic angle spectra, which are independent of the photoelectron angular distribution, were synthesised from these polarisation dependent spectra, as described previously.^8^

The liquid sample of pyrrole (CAS: 109-97-7, Sigma Aldrich, ≥99.5%) was subjected to several freeze–pump–thaw cycles and introduced as a vapour into the interaction region at room temperature.

All the energy calibrations were carried out using a mixture of either pyrrole and CO_2_ or pyrrole and N_2_. The photon energy scale of the total ion yield of pyrrole, in the vicinity of the C and N edges, was established by recording yields of the two mixtures, and using the known energies of the calibrant absorption features.^9,10^ The ionisation energy scales of the C and N core level photoelectron spectra of pyrrole were established using a similar procedure, and calibrated against the known 1s(C) and 1s(N) ionisation energies in CO_2_ (ref. 11) and N_2_ respectively.^12^ The energy scale of the valence shell photoelectron spectrum was established using the known D_0_ (X̃^2^A_2_) state adiabatic ionisation energy of 8.2099 eV of pyrrole.^13^ Autoionisation of the core excited neutral states lying in the vicinity of the C edge in pyrrole strongly affected the vibrational envelope of the D_0_ state photoelectron band to the extent that the peak associated with the adiabatic ionisation energy could not be identified. In contrast, the effect of autoionisation on the vibrational envelope of the D_1_ (Ã^2^B_1_) state photoelectron band was much smaller. Therefore, the electron kinetic energy scale of a resonantly excited Auger spectrum was determined by combining the known photon energy (from the calibrated total ion yield spectrum) with the ionisation energy of 9.2 eV for the prominent peak in the D_1_ (Ã^2^B_1_) state photoelectron band.^14,15^ This procedure was also applied to the resonantly excited Auger spectra recorded in the vicinity of the N edge of pyrrole.

### Computational methods

The ground-state geometry of pyrrole was optimised at the MP2/aug-cc-pVTZ level of theory. No imaginary frequencies were found, confirming that the structure corresponds to the minimum on the potential energy surface. This geometry was used as the basis for the simulated spectra and is reported in the SI Table S1. Various computational methods were used for the X-ray spectra simulations and benchmark calculations. Unless stated otherwise, the Q-Chem v. 6.2 ^16^ and OpenMolcas^17^ software packages were employed.

**Fig. 2:**
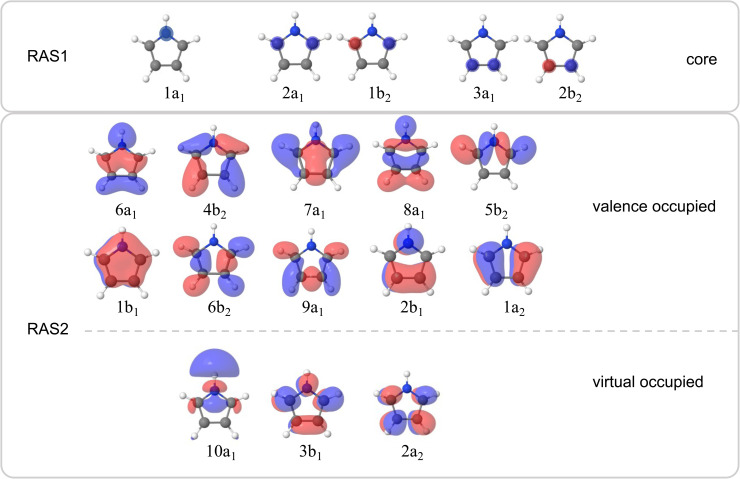
Active space orbitals involved in the RASPT2 calculations. The RAS1 space for the nitrogen K-edge spectra contained the 1a_1_ orbital, for the carbon 1s(C2, C3) spectra it contained the 2a_1_ and 1b_2_ orbitals, and for the carbon 1s(C4, C5) spectra it contained the 3a_1_ and 2b_2_ orbitals. The RAS2 space included the valence occupied and virtual orbitals shown in the figure. The RAS3 space was kept consistently empty.

The valence band photoelectron spectra were simulated using the EOM-IP-CCSD/aug-cc-pVTZ level and multi-reference calculations (RASPT2(19/14)) with aug-cc-pVTZ and ANO-L-TZVP basis sets, as implemented in Q-Chem v. 6.2 ^16^ and OpenMolcas^17^ software packages. In the RASPT2 calculations, the active space contained 19 electrons in 14 orbitals in the RAS2 space. The respective occupied orbitals are presented in Fig. 2. The virtual orbital space in the RAS2 space contained the orbitals shown in Fig. 2 plus one extra virtual orbital of A_1_ symmetry with Rydberg character to achieve a slightly better accuracy. The calculations were also benchmarked using various basis sets and other methods as shown in the SI. The intensities of the valence band photoelectron peaks were estimated as the norms of the respective Dyson orbitals in the EOM-IP-CCSD or RASPT2 approaches.

Core-level X-ray photoelectron spectra (XPS) were simulated using the fc-CVS-EOM-IP-CCSD^18^ approach and compared to those using the effective Maximum Overlap Method (MOM)^19^ with various basis sets.

X-ray absorption spectra (XAS) were simulated using the fc-CVS-EE-CCSD(fT) method^20^ with either the uncontracted u6-311++G(2p,2d) or the aug-cc-pVTZ basis set as in calculations of the valence band photoelectron spectra. The multireference RASPT2 calculations were also carried out using the active space with 9 (for nitrogen K-edge spectra) or 10 (for carbon K-edge spectra) occupied and 3 virtual orbitals in the RAS2 space (see Fig. 2). The RAS1 space contained either the 1s(N) orbital for calculations at the nitrogen K-edge, or the 1s(C2, C3), or 1s(C4, C5) orbitals for the carbon K-edge, resulting in RASPT2(20/14) for the nitrogen edge, and RASPT2(24/15) for the carbon edge which provided optimal convergence. In all the RASPT2 calculations, the RAS1 space was restricted to single electron occupation, and the RAS3 space was kept consistently empty. The ‘highly excited state’ (HEXS) scheme^21^ was employed to isolate the core-excited states by projecting out undesired valence excitations. An imaginary level shift of 0.25 Hartree was applied to avoid intruder state problems in the multistate RASPT2 (MS-RASPT2) treatment. In all cases, the Cholesky localisation scheme was used. For benchmarking purposes, the EOM-CCSD and RASPT2 methods were compared to the computationally more efficient tailored TDDFT with the SRC1-R1 functional.^22,23^

Resonant Auger spectra were simulated using the multireference RASPT2 method within the one-center approximation (OCA)^24^ as implemented in OpenMolcas.^17^ Alternatively, the Feshbach–Fano approach^25,26^ was employed, as implemented in Q-Chem v. 6.2.^16^ The initial core-excited states were described using the fc-CVS-EOM-EE-CCSD method, and the final states were treated at the EOM-IP-CCSD level. The continuum orbital was approximated with a plane wave and the integration was carried out for Lebedev's quadrature with the 5 order. While the final states corresponding to participator decay channels (1-hole, 1*h*) are well described by EOM-IP-CCSD, the accurate description of spectator channels (2-hole–1-particle, 2*h*1*p*) requires inclusion of triple excitations, which was achieved at the EOM-IP-CCSD(fT) level. The resulting energies and partial decay widths were convoluted with a Gaussian function of 0.2 eV full width at half maximum (FWHM).

Vibrationally resolved XAS spectra were modeled using an in-house program based on fitted potential energy surfaces for the ground, intermediate core-excited, and final ionised states calculated at the computationally effective and stable SRC1-R1/aug-cc-pVTZ level. The method employs a time-independent model in which the RAES intensities for bound-to-bound transitions are given by products of Franck–Condon factors (FCFs) between the respective states. Further methodological details are provided in the SI.

## Results and discussion

A complete interpretation of the molecular resonant Auger spectra requires an understanding of all the steps involved in the resonant Auger process itself. Therefore, we begin by examining the contributions to the X-ray absorption spectra at both the carbon and nitrogen K-edges to identify which core-excited states are accessible at selected photon energies. We then analyse the evolution of the Auger signal, as a function of the incoming photon energy, in terms of the contributions from both the participator and spectator processes. We also compare the Auger signal to the valence photoelectron spectrum, since the final ionised states in both processes are identical (see Fig. 1). The relative intensities of the Auger signal and the direct valence photoelectron signal differ because, in the Auger process, the transition probabilities are governed by the decay matrix elements from specific core-excited states, rather than by the direct photoionisation cross sections of the valence orbitals. Nevertheless, their comparison often provides a convenient guide for navigating the structure of the Auger signal.

For a complete discussion for the carbon RAES data, including symmetry-breaking dynamics, we constructed a linear vibronic coupling model which allowed the influence of the ultrafast core–hole dynamics on the final spectroscopic observable in the Auger signal to be assessed.

### X-ray absorption spectroscopy (XAS)

**Fig. 3:**
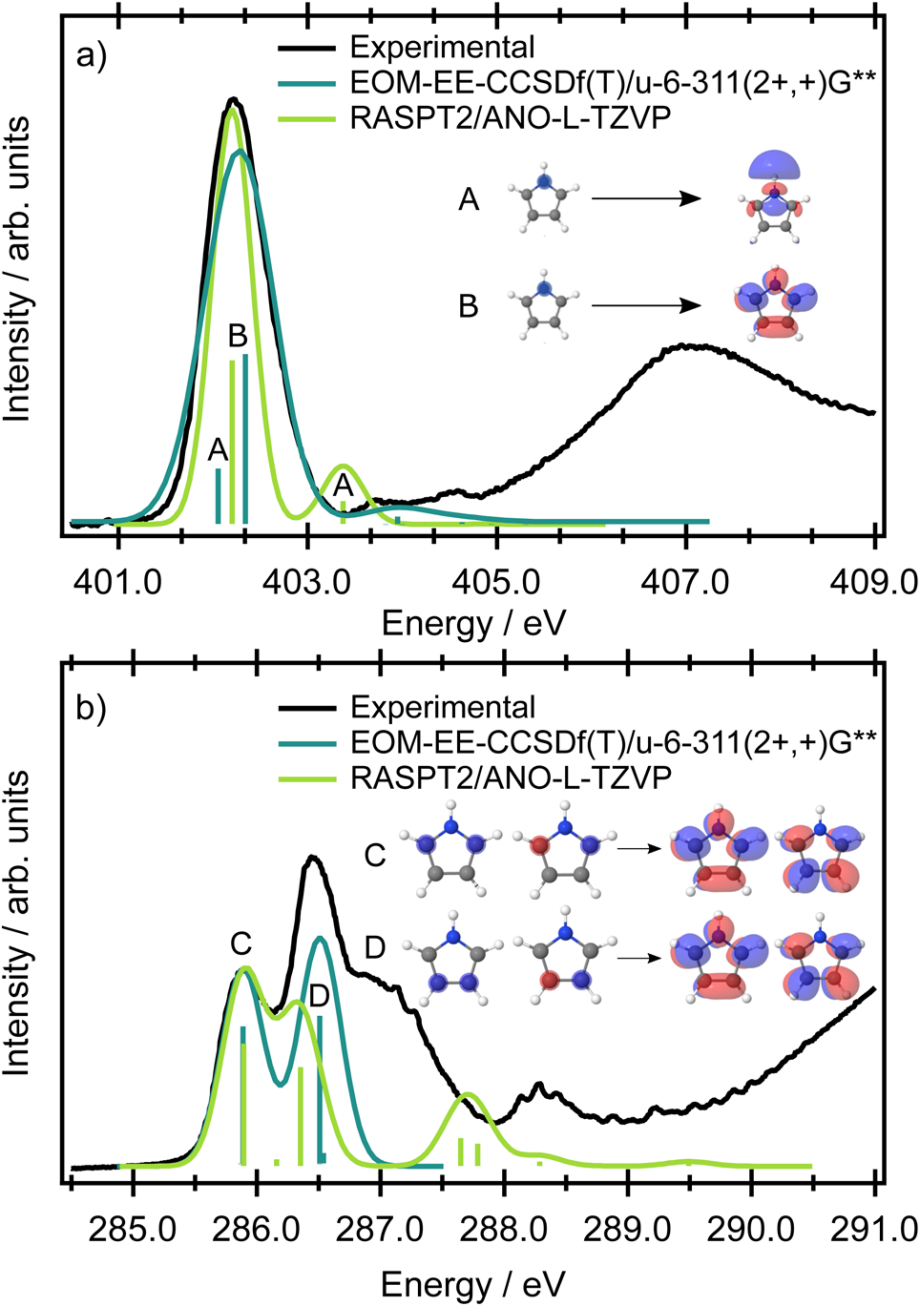
Nitrogen (panel a) and carbon (panel b) K-edge absorption spectra of pyrrole with vertical excitation energies calculated at the CVS-EOM-EE-CCSD(fT)/u-6-311(2+,+)G** and RASPT2/ANO-L-TZVP levels of theory. The calculated spectra have been broadened with a Gaussian lineshape with a FWHM of 0.2 and 0.4 eV for carbon and nitrogen, respectively. Energetic shifts of −1.12 eV (EOM) and 0.91 eV (RASPT2) have been applied for carbon, and of −1.35 eV (EOM) and −0.00 eV (RASPT2) for nitrogen, to align the lowest energy peak in the theoretical spectrum with the corresponding peak in the experimental spectrum.

The nitrogen 1s binding energy derived from XPS (Fig. S1 in the SI) is 406.1 eV; the calculated values, collected in Table S2 in the SI, are in excellent agreement with the experimental data. The experimental pre-edge XAS spectrum in Fig. 3 is dominated by a single, broad band centred at 402.28 eV with a width of 0.76 eV (FWHM). This line width is significantly broader than the experimental resolution (∼38 meV), even though the band exhibits no apparent shoulders or additional features. A computational analysis of this intense pre-edge feature suggests that the broad peak arises from two transitions, separated by a few tenths of an eV (depending on the method and basis set, see Table S3 in the SI), originating from the 1s(N) → σ* + 3pσ (10a_1_) Rydberg-valence and 1s(N) → π* (3b_1_) transitions. The respective orbitals are depicted in the inset of Fig. 3. A previous gas phase study on pyrrole reports excitations of the same character,^27^ and similar transitions have been reported for indole, which possesses a pyrrole ring as a subunit.^28^ The information in the XAS spectrum reflects the electronic structure of the valence states in pyrrole, in which the nitrogen contributes its lone pair to the aromatic π system. At the same time, the ring possesses energetically low-lying σ* orbitals, which are close in energy to the π* orbitals, and therefore states of both π* and σ* character are populated within the same energy range. All theoretical methods predict the most intense transition to be 1s(N) → π*(3b_1_) and the weaker transition to be 1s(N) → σ* + 3pσ. However, as the energetic ordering and intensities of these two transitions are dependent on the level of theory used, and the individual contributions are not resolved in the experimental spectrum, the ordering of the states is ambiguous (while EOM-EE-CCSD predicts the state having σ* + 3pσ character to be the lower energy state, RASPT2/ANO-L-TZVP predicts the lower energy state to have π* character, see also Table S3 in the SI) and cannot be resolved in XAS. In the energetic region approaching the ionisation threshold, there are several, theoretically predicted, less intense features of mixed σ* and Rydberg character (Fig. 3).

**Fig. 4:**
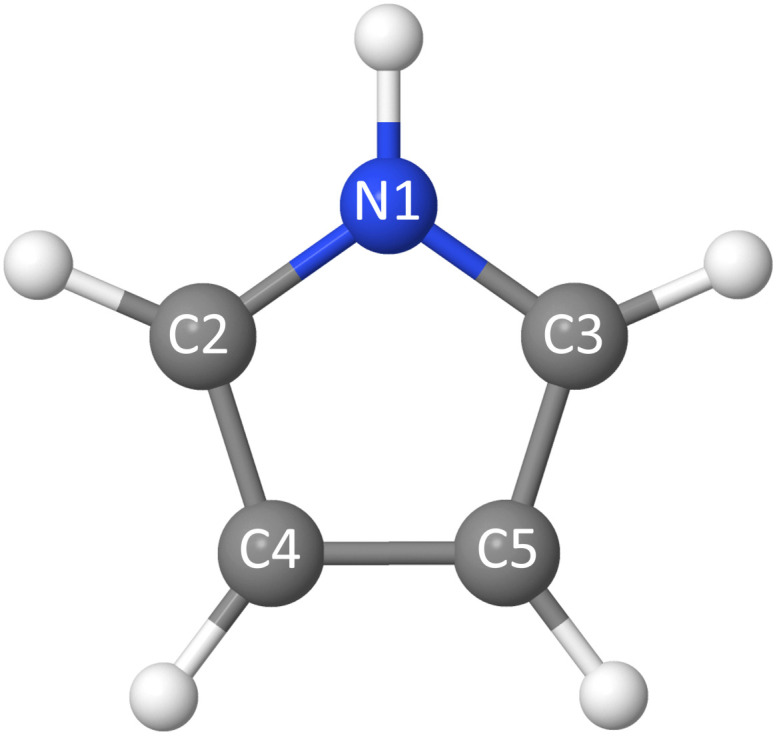
Pyrrole molecule with the atom numbering used in the manuscript.

The presence of two chemically-inequivalent carbon sites in pyrrole means that the pre-edge region of the carbon XAS spectrum is significantly more congested. The ground state minimum energy geometry of pyrrole is planar (*C*_2v_ symmetry point group) with two carbon chemical environments (Fig. 4) where C2, C3 are the carbons directly bonded to the nitrogen heteroatom and C4, C5 are furthest away. The presence of the two inequivalent carbon sites is reflected in the carbon XPS spectrum (Fig. S1 in the SI). The spectrum contains two bands, each of which exhibits vibrational structure. The lower energy band, with an adiabatic ionisation energy of 289.74 eV, corresponds to ionisation of the 1s (C4, C5) 3a_1_ and 2b_1_ orbitals, while the higher energy band, with an adiabatic ionisation energy of 290.69 eV, corresponds to ionisation of the 1s (C2, C3) 2a_1_ and 1b_1_ orbitals. Theoretically predicted ionisation energies, collected in Table S2, confirm the assignment.

The experimental carbon K-edge XAS spectrum (Fig. 3) contains two peaks, centred at 285.90 and 286.47 eV, the latter of which has a significant high energy shoulder. The energetic ordering of the core-excited states should follow the common trends in heterocycles – the excitation energy increases with the increasing electronegativity of the atom neighboring the carbon atom. The peak at lower energy (285.90 eV) corresponds to the transition from carbon atoms C4, C5 distant from the heteroatom, and the peak at 286.47 eV corresponds to the transition from the closer carbon atoms C2, C3.

The modelled XAS spectrum at the carbon K-edge reflects the presence of multicentre core orbitals. In the Franck-Condon point, the symmetric (2a_1_ and 3a_1_) and antisymmetric (1b_2_ and 2b_2_) linear combinations of the atomic 1s core orbitals for each carbon environment give rise to a set of degenerate A_2_ and B_1_ core-excited states. The states of B_1_ character are electric-dipole allowed and correspond to the mixed transitions 1s(C4, C5) (3a_1_) → π*(3b_1_) + 1s(C4, C5) (2b_2_) → π*(2a_2_) and 1s(C2, C3) (2a_1_) → π*(3b_1_) + 1s(C4, C5) (2b_2_) → π*(2a_2_). The states of A_2_ character are forbidden and correspond to the mixed transitions 1s(C4, C5) (2b_2_) → π*(3b_1_) + 1s(C4, C5) (2a_1_) → π*(2a_2_) and 1s(C2, C3) (2b_2_) → π*(3b_1_) + 1s(C2, C3) (2a_1_) → π*(2a_2_). In the following text, we use a shorter notation 1s(C2, C3) → π* *etc.* to denote these transitions. The transition energies at the fc-CVS-EOM-EE-CCSD and RASPT2 levels are shown in Fig. 3, and the benchmark calculations are presented in Table S3 in the SI. All the theoretical methods (ranging from CVS-ADC(2)-X to CVS-EOM-EE-CCSD or TDDFT, with the tailored short-range functional SRC1-R1) predict transitions from 1s(C2, C3) to be as probable as those from 1s(C4, C5).

According to the EOM-CCSD calculations, the shoulder evident in the experimental XAS spectrum at ∼287 eV corresponds to the transitions from the 1s(C4, C5) carbon orbitals to the σ* virtual orbitals. In the RASPT2 calculations, these transitions were not observed since the active space contained only a limited number of virtual σ* orbitals.

From the simple comparison between the experimental spectrum and the theoretical calculations it is clear that consideration of only the vertical electronic transitions from a perfectly symmetric minimum energy structure does not reproduce many of the prominent features observed in the carbon XAS spectrum.

For many symmetric molecules, core-exited states can couple vibronically which leads to the so-called symmetry-breaking process (or dynamical core–hole localisation).^29–33^ This process has been observed in several systems such as pyrazine.^34–37^ This vibronic coupling dynamically lifts the degeneracy between the symmetry-adapted core-excited configurations and drives localisation of the core hole on one atomic site. As a result, the molecular symmetry is effectively reduced (from *C*_2v_ to *C*_s_), leading to intensity borrowing between symmetry-allowed and formally forbidden transitions and to the appearance of closely-spaced peaks in X-ray absorption spectra.

**Fig. 5:**
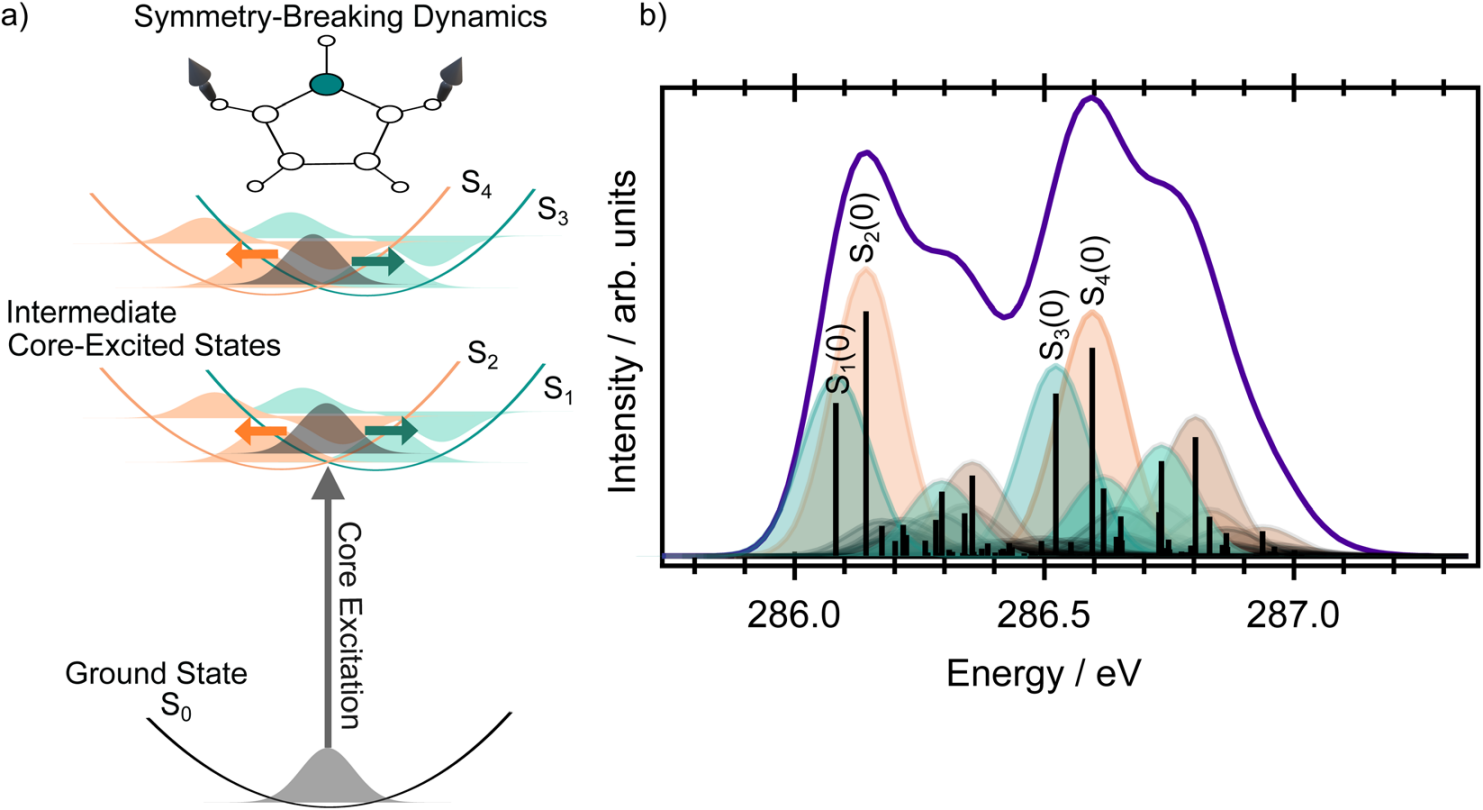
(Panel a) Sketch showing cuts through the diabatic potential energy surfaces along one of the vibrational normal modes that drives the symmetry breaking in the core-excited states. After the core-excitation, the wave packet can evolve from the Frack–Condon point on both diabatic states S_1_ and S_2_ (or S_3_ and S_4_) with equal probability. (Panel b) Vibrationally resolved X-ray absorption spectrum at the carbon K-edge modelled within the LVC model based on potential energy cuts calculated at the SRC1-R1/aug-cc-pVTZ level.

In our study, we constructed a linear vibronic coupling (LVC) model based on one-dimensional cuts of the potential energy surfaces along selected normal modes. The potential energy surfaces, computed at the SRC1-R1/aug-cc-pVTZ level of theory, were fitted to polynomial functions. Full details of the time-independent model based on the Franck–Condon interference approach introduced by Neeb *et al.*^38^ are provided in the SI. The core-excited states (the adiabatic A_2_ and B_1_ states) were represented in a diabatic basis, where the two surfaces cross in the Franck–Condon region (the coupling constant between the diabatic surfaces is very small). This crossing reflects a situation in which the core hole is localised either on the left or on the right side of the molecule, and both states are equally accessible due to an equivalent transition dipole moment in the minimum of the potential energy curve (see Fig. 5). The coupling between the respective diabatic states is very small, with the coupling constant *λ* estimated to be less than 0.03 eV. Due to the coupling, the zero point energies of the diabatic states S_1_ and S_2_ (for core excitation from carbons C4, C5) and S_3_ and S_4_ (for core excitation from C2, C3) differ by 0.06 eV, which may be slightly overestimated (see also Fig. 5).

The resulting XAS spectrum, shown in Fig. 5, reproduces the overall experimental shape well. Due to symmetry-breaking dynamics, both diabatic states S_1_ and S_2_, and S_3_ and S_4_, corresponding to the two inequivalent carbon environments, become populated in the XAS spectrum (see labels in Fig. 5). Each state exhibits a vibronic progression, primarily driven by low-frequency ring-puckering modes (the respective vibrational normal modes are depicted in Fig. S2, the potential energy cuts along the normal modes are shown in Fig. S3, and the excitation energies are listed in Table S5 in the SI) contributing to the width of the experimental peaks. Based on the data, we can also argue that the pronounced shoulder at ∼287 eV is a convolution of the vibrational signal and the electronic transitions to the σ* virtual orbitals.

In the experimental XAS spectra, the vibrational structures are obscured by the broadening associated with the short core–hole lifetime (∼80 meV for carbon^39^), and thus individual progressions cannot be resolved and may seem irrelevant for spectral interpretation. However, photoexcitation of the core–hole states is the first step in the resonant Auger process, and, under the experimental conditions employed in the present experiment where the photon bandwidth is narrower than the core–hole lifetime (resonant Raman conditions^40^), only a limited set of vibrational states is selectively excited. This, in turn, is reflected in the changing shape of the Auger spectrum. Hence, the correct description of the photoexcitation process is crucial not only for interpretation of the XAS spectrum but also for RAES.

The ambiguity in assigning the electronic states at the nitrogen K-edge, and the intricate convoluted vibronic structure at the carbon edge, result in non-trivial XAS spectra that are difficult to interpret experimentally—especially in the absence of supporting theory. As we discuss below, the RAES signal is not core–hole lifetime limited and therefore offers additional and complementary information about the electronic structure and the nuclear dynamics in the excited states crucial for interpreting the XAS spectra.

We begin our analysis by interpreting the nitrogen resonant Auger spectra. In this case, the Auger signal helps to unambiguously disentangle which electronic states contribute to the XAS spectrum. This analysis provides a foundation for understanding the more complex carbon resonant Auger spectra, in which the Auger signal contains not only information about the contributions of electronic states but also information on the symmetry-breaking nuclear dynamics in the intermediate core-excited states.

### Resonant Auger spectroscopy

All the resonant Auger electron spectra in this work were recorded under the resonant Raman conditions (the incident photon energy bandwidth of ∼40 meV is narrower than the natural line widths of ∼80 meV for carbon 1s and ∼110 meV for nitrogen 1s). Therefore, by tuning the photon energy across an absorption resonance, the resulting Auger spectra have sensitivity to the relative contributions of multiple transitions that cannot be resolved in an X-ray absorption spectrum.^40^

In the resonant Auger spectra, two distinct types of decay channels contributing to the spectrum can be identified: participator and spectator channels, see Fig. 1. In a participator channel, the initially excited electron is involved in the core–hole recombination, resulting in a final 1*h* state with a hole in one of the valence orbitals. The signal corresponding to these channels appears in the high kinetic energy region in the RAES and energetically coincides with that from states accessed *via* direct valence photoionisation, as both processes result in the same 1*h* electronic configurations. This overlap allows the direct photoionisation signal to serve as a reference for assigning participator channels.

The signal due to the spectator channels in RAES appears at lower kinetic energies and corresponds to final states in which the valence orbitals contain two holes, while the initially excited electron remains in a virtual orbital. The spectator channels dominate the total RAES signal at lower kinetic energies as there are a greater number of possible electronic configurations achievable from a spectator process, and so the density of the spectator states becomes much higher than that of the participator states. Thus, the assignment of spectator states is more challenging, both experimentally and theoretically, owing to the complex nature of the electronic structure of the final states and their broad spectral distribution. As a result, the straightforward qualitative interpretation of the spectator peaks can be less amenable but as the intensities of the participator and spectator processes are governed by different propensity rules, the spectator features can provide important, complementary information on the electronic structure.

#### Nitrogen RAES

**Fig. 6:**
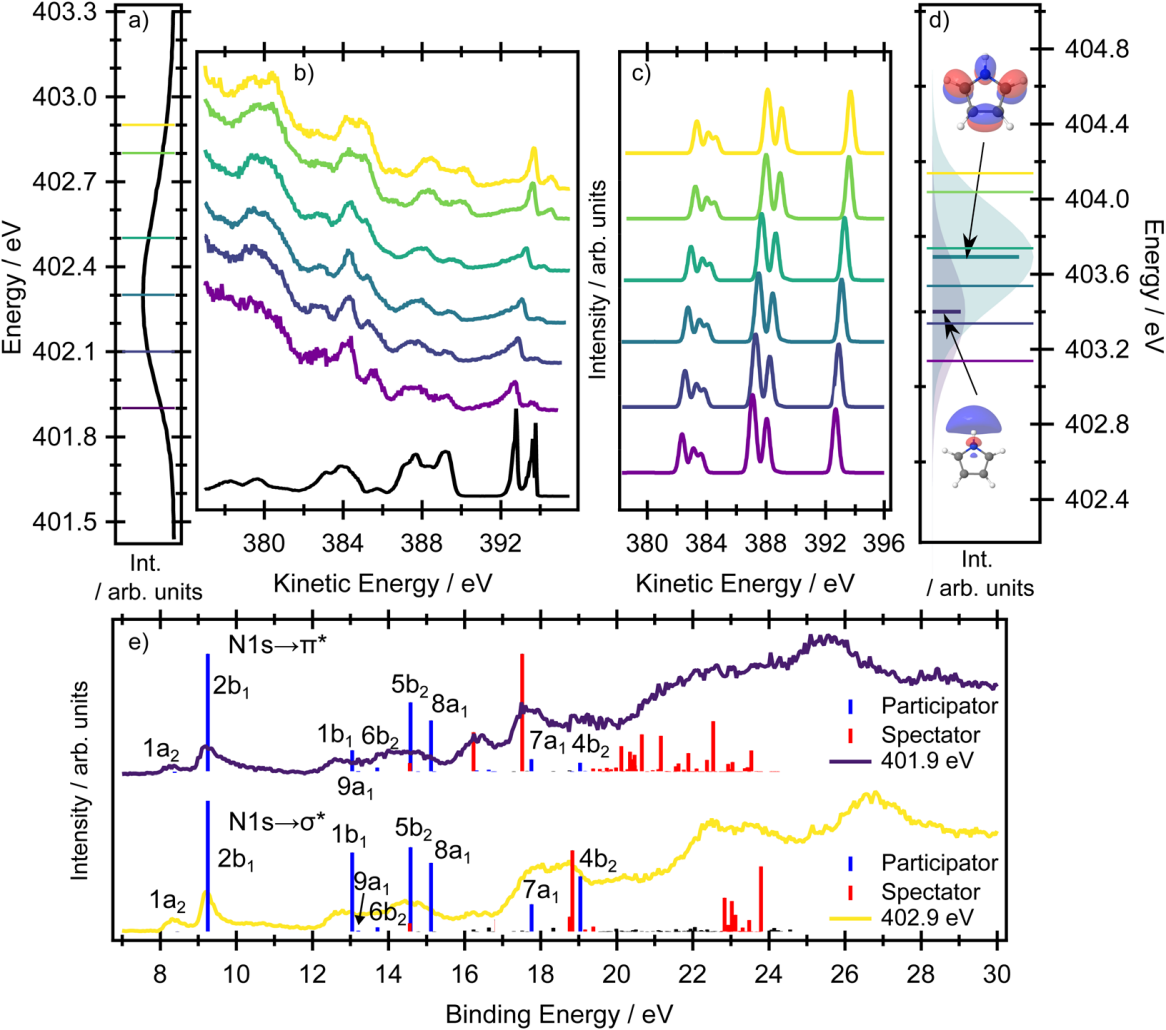
(Panel a) Experimental nitrogen K-edge XAS spectrum, and (panel b) resonantly excited nitrogen K-edge Auger spectra of pyrrole. The coloured lines on the XAS spectrum (a) indicate the central excitation energy used to record the resonant Auger spectra. The direct valence band photoelectron spectrum, recorded at a photon energy of 60 eV, is included in black, with an adjusted electron kinetic energy scale to facilitate comparison with the resonant Auger spectra. (Panels c and d) Calculated resonant Auger electron spectra and XAS spectra at the EOM-CCSD(fT)/u-6-311(2+,+)G** level. The thick lines in (panel d) represent the XAS spectra as sticks, prior to Gaussian broadening by a phenomenological 0.4 eV to facilitate comparison with the experimental spectrum. The thin coloured lines on the XAS spectrum in (panel d) indicate the energies used to calculate the resonant Auger spectra. The Auger signal is calculated as a weighted average between the 1s(N) → σ* and the 1s(N) → π* RAES signals, where the weight is given by the respective oscillator strength at a given photon energy. (Panel e) Experimental and calculated RAES spectra at the RASPT2/ANO-L-TZVP level for the 1s(N) → σ* (yellow) and 1s(N) → π* (purple) resonances (Tables S8 and S9). The binding energy of the simulations was adjusted so that the ionisation energy of the 2b_1_ orbital was 9.2 eV.

The first resonance in the XAS spectrum at the nitrogen K-edge has heavily overlapped contributions from the 1s(N) → σ* and 1s(N) → π* transitions (Fig. 6) and, at most of the photon energies used to record the RAES spectra, some contribution from each electronic state is excited (Fig. 6d) showing the theoretical spectra with incident photon energies). As a result, the participator bands in the resonantly excited Auger spectra show only a limited evolution as a function of photon energy.

The two bands observed at the highest kinetic energies in Fig. 6 (above ∼392 eV) correspond to ionisation from the two highest occupied orbitals 1a_2_ (HOMO) and 2b_1_ (HOMO−1), both of π character. The corresponding calculations of the resonant Auger signal at the EOM-CCSD(fT) level of theory are presented in Fig. 6c, with full transition lists and intensities included in the SI (Tables S6 and S7). The corresponding RASPT2/ANO-L-TZVP simulations are included in Fig. 6e, with further transition lists in the SI (Tables S8, S9, Fig. S4 and S6). Both electronic structure methods show an intense band associated with the 2b_1_ hole state but nearly complete suppression of the 1a_2_ hole state at all excitation energies. In the experimental spectrum, the weak feature that is an energetic match for the 1a_2_ hole state arises from a small contribution from direct photoionisation, which has a low, but non-zero, cross-section at these photon energies.

At the highest incident photon energies, the relative intensity of the 1*h* state associated with the 1a_2_ orbital continues to increase. In a very simplified picture, the intensity of a participator process would depend on the spatial overlap between the core hole with the final 1*h* state. For a qualitative understanding it is possible to use population analysis schemes (such as the Löwdin population scheme)^41^ or to consider the spatial overlap of the orbitals involved in the resonant Auger process. The orbitals representing the final 1*h* states are the same as the valence orbitals depicted in Fig. 2, which we can use for a qualitative discussion. For example, it is evident that there is no spatial overlap between the 1s(N) orbital and the 1a_2_ orbital, for which all of the electron density is centred on the carbon sites. Therefore the intensity of the participator channel leading to the respective 1*h* state (1a_2_ participator channel, D_0_ state) is very weak. This is in contrast with the D_1_ state, where the final state involves an orbital of 2b_1_ symmetry localised on the 1s(N) site. The same qualitative interpretation of the participator channels holds for both the 1s(N) → σ* and the 1s(N) → π* transitions. This insensitivity to the excitation process results in the participator bands being similar for both the 1s(N) → σ* (^1^A_1_) and the 1s(N) → π* (^1^B_1_) core-excited states. It also means that the Auger signal shows little variation with photon energy.

Overall, the agreement between the EOM-CCSD(fT) simulations and the experimental spectra is excellent for the peaks in the high kinetic energy region. In the energy region between 387–390 eV the simulations capture the dominant spectral features but provide less accurate results for the final state in which the hole is localised in the 1b_1_ orbital, as this orbital cannot be described accurately with a single-reference method. The breakdown of the single-particle approximation also affects the valence band photoelectron spectrum,^42^ also see a benchmark Table S18 in the SI. For this kinetic energy region of the experimental spectra (corresponding to the 12–16 eV binding energy region), the multiconfigurational RASPT2 method provides more accurate results (see Fig. 6e). As can be seen from the RASPT2 spectra, the agreement between the calculated and experimental spectra in the region attributed predominantly to participator channels is excellent.

At kinetic energies lower than 385 eV (Fig. 6b), spectator processes are now energetically feasible and are therefore expected to make more significant contributions. Accordingly, the experimental spectra recorded at different photon energies, in which the ratio of the initially excited states corresponding to the 1s(N) → σ* and 1s(N) → π* transitions varies, show the greatest sensitivity to the initially prepared states. Indeed, this region in the experimental spectra is clearly composed of multiple contributions which show different excitation energy dependencies in their intensities. At lower photon energies (*e.g.* 401.9 eV), there is a strong peak at 385.5 eV kinetic energy and a pair of well-resolved features at 384.1 and 382.7 eV. Comparing this spectrum to that recorded at a higher incident photon energy (*e.g.* 402.9 eV) shows a suppression of the feature at 385.5 eV and, in a similar energetic region to the pair of peaks mentioned previously, the appearance of a broad band with almost a ‘flat top’ profile. The RASPT2/ANO-L-TZVP calculations presented in Fig. 6e (binding energy region above 16 eV) show two well-resolved and intense spectator features for the 1s(N) → π* resonance which correspond very well with the structure observed in the experimental spectrum. The spectator channels correspond to the final ionised state with one hole in the 2b_1_ orbital, another hole in the 1b_1_ orbital, and an electron in the 3b_1_ orbital (and partially in the 2a_2_ orbital, as the corresponding wave function is heavily mixed). In comparison, for the 1s(N) → σ* resonance, the most intense spectator feature occurs around 18.5 eV and corresponds to a heavily mixed wave function with two holes in the highest occupied valence orbitals and an electron in the 3b_1_ orbital (and partially in the 2a_2_ orbital). This peak contributes to the broad band observed for the 1s(N) → σ* resonance.

Overall, the RASPT2-calculated Auger spectra successfully reproduce the excitation energy dependent intensity changes associated with the 1s(N) → σ* and 1s(N) → π* transitions. Agreement between the experimental and calculated RAES spectra further relies on a correct energetic ordering of these core-excited states, with the 1s(N) → σ* transition lying below the 1s(N) → π* transition. This ordering is correctly predicted by RASPT2 (although the energy splitting is overestimated), whereas it is not reproduced by the EOM-CCSD approach.

Clearly, the spectator features are sensitive to the 1s(N) → σ* or 1s(N) → π* transitions. While the degree of orbital localisation on the nitrogen site is similar for both transitions, the 1s(N) → π* transition involves the delocalised π* orbital on the ring system. The final Auger states producing electrons with kinetic energies below 390 eV all involve ring-based orbitals. This dependence on the final orbital for the spectator peaks means they can be used to distinguish between the preparation of the core-excited state through the 1s(N) → σ* or the 1s(N) → π* transitions. The RAES data can therefore be used to unambiguously assign the state ordering, which cannot be achieved only with quantum chemical methods and the XAS spectra alone.

#### Carbon RAES

**Fig. 7:**
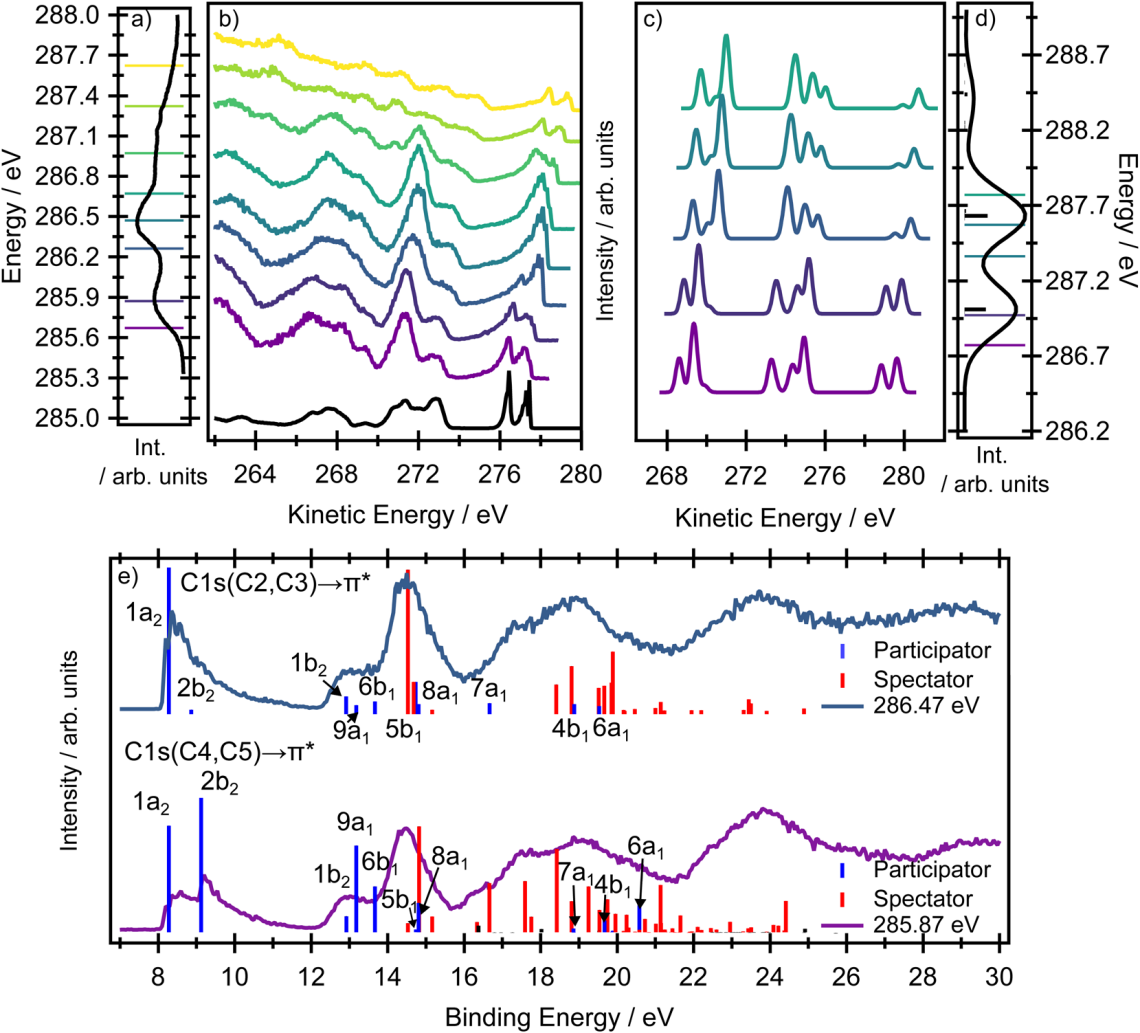
(Panel a) Experimental carbon K-edge XAS spectrum and (panel b) the resonantly excited Auger spectra of pyrrole. The coloured lines on the XAS spectrum (a) indicate the central excitation energy used to record the resonant Auger spectra. The direct valence band photoelectron spectrum, recorded at a photon energy of 60 eV, is included in black, with an adjusted electron kinetic energy scale to facilitate comparison with the resonant Auger spectra. (Panels c and d) calculated resonant Auger electron spectra and XAS spectra at the EOM-CCSD(fT)/u-6-311(2+,+)G** level. The thick lines in (panel d) represent the XAS spectra as sticks, prior to Gaussian broadening by a phenomenological 0.2 eV to facilitate comparison with the experimental spectrum. The thin coloured lines on the XAS spectrum in (panel d) indicate the energies used to calculate the resonant Auger spectra. The Auger signal is calculated as a weighted average between the 1s(C4, C5) → π* and the 1s(C2, C3) → π* RAES signals, where the weight is given by the respective oscillator strength at a given photon energy. (Panel e) Calculated RAES spectra at the RASPT2/ANO-L-TZVP level for the 1s(C4, C5) → π* (purple) and 1s(C2, C3) → π* (blue) resonances (Tables S12 and S13). The binding energy of the simulations was adjusted so that the ionisation energy of the 2b_1_ orbital was 9.2 eV.

While the photoelectron bands associated with the participator decay channels in the resonantly excited nitrogen K-edge Auger spectra exhibited only a minimal evolution as the photon energy was scanned across the absorption resonance, the resonantly excited Auger spectra at the carbon K-edge show very pronounced changes in both the participator and the spectator channels (Fig. 7).

At the two highest photon energies, most of the spectral signal comes from direct ionisation with only a small portion from autoionisation, and we therefore focus the discussion mainly on the signal recorded at lower photon energies.

**Fig. 8:**
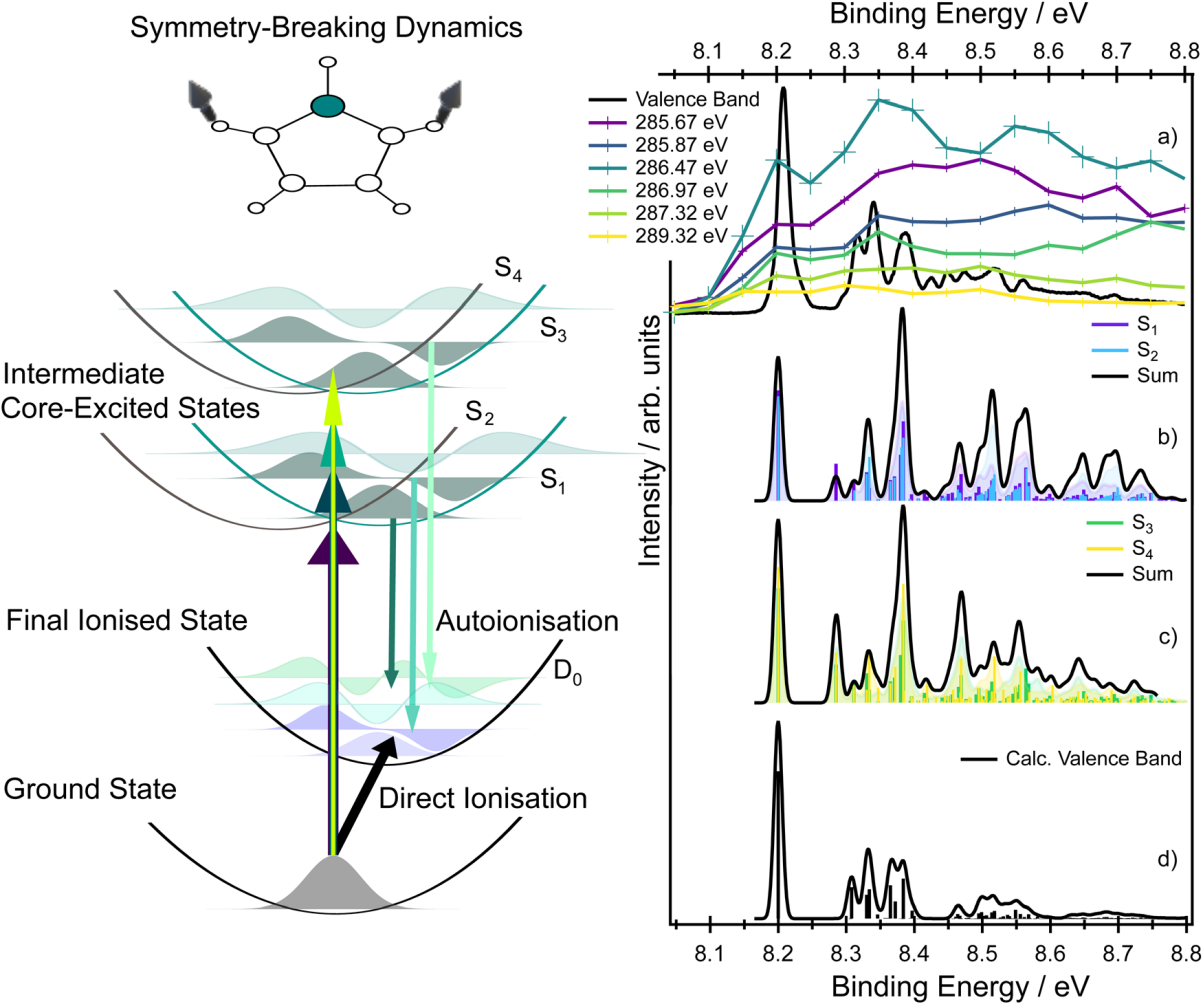
Left panel: sketch of core-excitation of the two inequivalent carbon sites connected with symmetry-breaking dynamics in the intermediate core-excited states along a symmetry-breaking vibrational coordinate. (Panel a) Experimental vibrationally resolved RAES measured at various photon energies compared to the vibrationally resolved photoelectron spectrum of the D_0_ state due to direct ionisation using a photon energy of 30 eV. Theoretically modelled vibrational progressions seen in RAES for the D_0_ state if approached through S_1_ and S_2_ (panel b) or S_3_ and S_4_ (panel c). (Panel d) Simulated vibrationally resolved photoelectron spectrum of the D_0_ state (direct photoionisation).

A very notable change occurs in the kinetic energy range ∼276–278 eV. For example, the Auger spectra recorded at the lowest and highest photon energies (plotted in purple and blue, respectively, in Fig. 7) contain a distinct doublet at high kinetic energies, with the two peaks associated with the D_0_(^2^A_2_) and the D_1_(^2^B_1_) ionic states. However, at intermediate excitation energies, the doublet undergoes pronounced changes in the overall shape (see zoomed in picture in Fig. 8). As we show below, the peculiar spectral shapes are not dominantly due to changes in the relative contributions of the two electronic final states but to symmetry-breaking dynamics in the intermediate states.

In a purely electronic picture, excitation at photon energies below 286 eV corresponds to transitions originating from the C4, C5 sites while, above 286 eV, the dominating transition originates from the C2, C3 sites, the equivalent carbons closest to the nitrogen site. The theoretically predicted excitation energies are collected in Table S3 in the SI and all the calculations support this assignment. Both the RASTP2 and the fc-CVS-EOM-EE-CCSD(fT) calculations suggest that the two peaks in the doublet should undergo pronounced changes in their relative intensities as the excitation energy changes. The 2b_1_ orbital has negligible electron density localised on the C2, C3 sites. Thus, excitation from C2, C3 creates core holes with minimal overlap with the 2b_1_ orbital, and therefore the participator decay to the corresponding 1*h* final state (D_1_) will be weak. In contrast, excitation at lower photon energies creates core holes on the C4, C5 sites, and these overlap both the 1a_2_ and the 2b_1_ orbitals. Both the EOM-CCSD(fT) and the RASPT2 methods model this part of the RAES spectrum accurately.

In the electron kinetic energy range 271–273 eV in the experimental spectrum, the EOM-CCSD(fT) method (in the corresponding kinetic energy range 272–276 eV) provides a slightly poorer agreement with the experiment than does the RASPT2 method. Again, this is due to the breakdown of the one-particle model and to a large contribution of spectator states. In this case (binding energy region 12–16 eV), the RASPT2 results provide a superior quality description of the significant spectator channels (see Tables S12 and S13 in the SI for individual assignments, also see Fig. S8 and S10). The experimental electron kinetic energy range below 271 eV is dominated by spectator states and the RASPT2 calculations (in the binding energy above 16 eV) provide very good agreement with the experimental spectrum. Overall, while the purely electronic model shows a reasonable qualitative agreement with many of the trends observed in the experimental spectrum, the changing asymmetry in the peak shapes in the kinetic energy range ∼272–276 eV region cannot be fully reproduced (see a zoomed in picture in Fig. 8a).

To help understand the spectral shape associated with the D_0_ final state in the kinetic energy range ∼277–281 eV (8–9 eV in binding energies), we can use the sketch in Fig. 8. As was discussed previously, the intermediate core-excited states can couple vibronically, which leads to the intensity borrowing and observation of optically forbidden (in the Franck–Condon point) states in the XAS. In these circumstances, a narrow band excitation results in the population of a set of vibrational wave functions (a wavepacket) in the intermediate core-excited states of both S_1_ and S_2_ (or S_3_ and S_4_); the sketch shows for simplicity only vibrational wave functions of the S_1_ and S_3_ states. The potential energy surfaces corresponding to the S_1_ and S_2_ (and S_3_ and S_4_) states exhibit shifted minima with respect to the ground electronic state and importantly also with respect to the final ionised state D_0_. Therefore, projection of the vibrational wavepacket from the intermediate states to the final ionised state D_0_ clearly leads to a vibrational progression which differs from that due to direct photoionisation. The intermediate state does not simply act as a ‘mirror’, reflecting the vibrational wavepacket to the final state, but instead influences the shape of that wavepacket in a symmetry-breaking process. A comparison between the vibrational progressions of the D_0_ state formed after direct ionisation and those formed during autoionisation, at various incoming photon energies, is provided in Fig. 8a–d.

The LVC model introduced for modelling the X-ray absorption spectrum can, in principle, be extended to model the vibrationally-resolved RAES. The spectral weight of the final ionised states in RAES is determined by a multiplication of a dipole matrix element between the initial and the intermediate core-excited state (initial core–hole creation) by a Coulomb matrix element between the intermediate and the final ionised state (Auger decay). This final signal can be compared to the direct ionisation process.

Previous high resolution valence shell photoelectron spectra of pyrrole^14,15^ have revealed significant vibronic progressions in both the D_0_ and D_1_ states. For both states, however, the vibrationally resolved spectrum is always dominated by the adiabatic transition to the vibrationless level of the ionised state, and vibronic progressions are mainly due to the excitation of a single quantum in the ring deformation and ring puckering modes (ν_8_, ν_11_, ν_14_, ν_15_, ν_16_). The calculated vibrationally resolved spectra for the valence band photoelectron spectra are shown in Fig. 8d, and the assignments are provided in Table S19. However, the vibronic progressions significantly differ if the final state is reached *via* an intermediate state; see the comparison in Fig. 8b–d. The simulated vibrationally resolved RAES corresponding to the vibrational progressions from the S_1_ and S_2_ states, and the S_3_ and S_4_ states, to the ionised D_0_ state are shown in the same figure.

The simulations show that the adiabatic transition to the vibrationless level of the ionised state is not the most intense feature; instead, vibrational progressions associated with ring-deformation modes appear with significant intensity. The vibrational modes populated following direct ionisation differ from those accessed *via* autoionisation of the intermediate state, reflecting the previously described influence of symmetry breaking in the intermediate state and giving rise to new progressions involving the ν_4_, ν_12_, ν_17_, and ν_18_ modes (see Tables S4, S14–S17 in the SI).

The most important message from this analysis is that a detailed comparison between the vibrational envelope of an ionic state formed through resonant Auger decay and the corresponding envelope of the ionic state formed through direct ionisation provides clear evidence of symmetry-breaking intermediate state dynamics. Similar results have been reported previously for much smaller molecules, for which the RAES spectrum is more easily resolved.^38,43^ The change in the vibrational envelope observed in RAES, which is associated with the intermediate state dynamics, should be a general phenomenon for any symmetric molecule with a bound intermediate core-excited state and further investigation, potentially with time-dependent modelling, would be highly desirable.

## Conclusions

Resonant Auger electron spectroscopy is a complex but information-rich technique for molecular spectroscopy which offers a high degree of selectivity for understanding the electronic and nuclear dynamics of a molecule from complementary perspectives. The participator features can be readily assigned through comparison to valence photoelectron spectra obtained through direct ionisation. The intensity information in the participator spectrum provides a direct measure of the spatial overlap between the core hole and the final-state electron density, or, in other words, a measure of the amount of ‘atomic’ character in the final state. While modelling of the spectator decay is more complex and relies on the use of multi-reference methods, the spectator contributions are typically more intense than those due to participator decays, and provide an additional insight into the electronic configuration. The sensitivity of RAES to the dynamics in core-excited states is an additional aspect to the technique and our recent advances in combining high-resolution experimental measurements with the LVC models enable this information to be interpreted in terms of the nuclear motion of the molecule.

The full exploitation of some of the information in RAES requires performing high resolution measurements, both on the excitation and the detection side of the experiment. The development of higher photon-flux sources has been key for advancing resonant Auger measurements^44^ by enabling higher data acquisition rates, but one key challenge for all of the ‘photon-hungry’ resonant techniques, particularly when extending these methods to the time-domain, is the often poor monochromator transmission.^45^ For time-resolved measurements, the use of a monochromator introduces unwanted stretching of the pulse duration. Correlation-based approaches with noisy, broadband X-ray sources have been successful in recovering sub-bandwidth spectral information for a number of spectroscopic techniques, particularly absorption,^46–48^ though the application to resonant methods has been more limited.^45^ Being able to combine high-energy resolution and time resolution with resonant Auger probes would offer an exciting tool for probing both chemical dynamics and more fundamental questions around electronic structure.

## Author contributions

RAI, HMG, EM, and DMPH performed the experiment, with beamline support from JB and AM. RAI, HMG, and DMPH analyzed and interpreted the experimental data. EM developed the theoretical framework and performed the computational work with DN and ML. RAI, DMPH and EM conceptualised the project. All authors reviewed and approved the final version of the paper.

## Conflicts of interest

There are no conflicts to declare.

## Supplementary Material

SC-OLF-D5SC09051B-s001

## Data Availability

All experimental spectra and computational results are available at https://figshare.com/s/0eae30c0c1e405adecb0. Supplementary information (SI) is available. See DOI: https://doi.org/10.1039/d5sc09051b.
