# Supplementary material for: Ultrafast dynamics in core-excited states probed by resonant Auger spectroscopy: pyrrole
Source: Chem Sci. 2026 Feb 27;17(15):7773–86. doi: 10.1039/d5sc09051b (PMC12946931; doi:10.1039/d5sc09051b)
Supplement: SC-017-D5SC09051B-s001 [file SC-017-D5SC09051B-s001.pdf]

Supplementary Information (SI) for Chemical Science.
This journal is © The Royal Society of Chemistry 2026

Ultrafast dynamics in core-excited states probed by resonant Auger spectroscopy: Pyrrole

D. M. P. Holland,[1] H. G. McGhee,[2] M. Lamanec,[3,4] D. Nachtigallová,[3,4] A. Milosavljević,[5] J. D. Bozek,[5] E. Muchová,[6] and R. A. Ingle[7]

[1] *STFC, Daresbury Laboratory, Daresbury, Warrington, Cheshire WA4 4AD, United Kingdom.*

[2] *Department of Chemistry, 20 Gordon Street, London, WC1H 0AJ, United Kingdom.*

[3] *Institute of Organic Chemistry and Biochemistry, Czech Academy of Sciences, Flemingovo náměstí 542/2, 16000 Prague, Czech Republic.*

[4] *IT4Innovations, VŠB-Technical University of Ostrava, 17. listopadu 2172/15, 70800 Ostrava-Poruba, Czech Republic*

[5] *Synchrotron SOLEIL, L'Orme des Merisiers, Gif-sur-Yvette, 91192, France.*

[6] *Department of Physical Chemistry, University of Chemistry and Technology in Prague, Technická 5, Prague, 166 28, Czech Republic. E-mail: muchovae@vscht.cz*

[7] *Department of Chemistry, 20 Gordon Street, London, WC1H 0AJ, United Kingdom. E-mail: r.ingle@ucl.ac.uk*

STRUCTURAL PARAMETERS

Atom	X (Å)	Y (Å)	Z (Å)
N	0.0000	1.1174	0.0000
C	1.1233	0.3345	0.0000
C	−1.1233	0.3345	0.0000
C	0.7084	−0.9847	0.0000
C	−0.7084	−0.9847	0.0000
H	0.0000	2.1225	0.0000
H	2.1073	0.7700	0.0000
H	−2.1073	0.7700	0.0000
H	1.3572	−1.8440	0.0000
H	−1.3572	−1.8440	0.0000

TABLE S1. Geometry of pyrrole in the ground electronic state optimized at the MP2/aug-cc-pVTZ level. Coordinates are in Å.

CORE-LEVEL X-RAY PHOTOELECTRON SPECTRA (XPS)

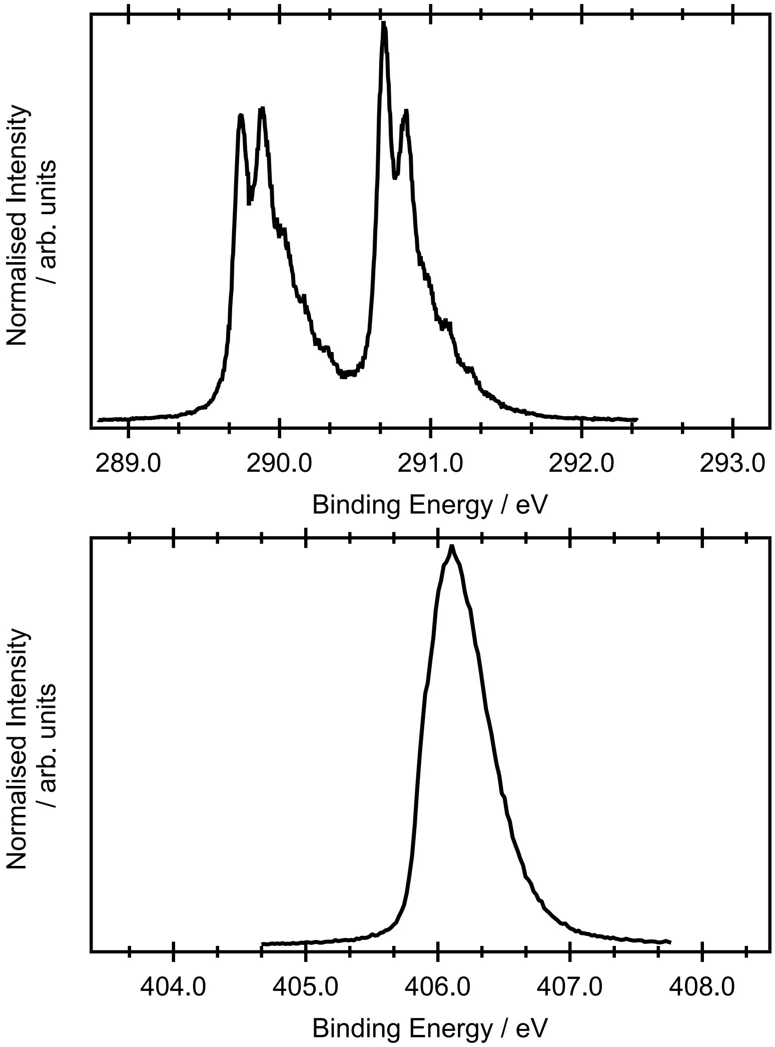

FIG. S1. $1s$(C) (upper panel) and $1s$(N) (lower panel) experimental XPS of pyrrole.

Carbon K-edge	1s(C4,C5)		1s(C2,C3)	
Method	Energy / eV	Intensity	Energy / eV	Intensity
CVS-EOM-IP-CCSD/aug-cc-pCVTZ	290.18, 290.14	0.8819, 0.88195	291.25, 291.25	0.88025, 0.88025
CVS-EOM-IP-CCSD/aug-cc-pVTZ	290.38, 290.34	0.8817, 0.8818	291.44, 291.44	0.88005, 0.88005
CVS-EOM-IP-CCSD/u-6-311(2+,+)G**	290.62, 290.66	0.8794, 0.8793	291.75, 291.75	0.87745, 0.87745
CCSD(T) MOM/aug-cc-pCVTZ	289.70, 289.74	-	290.68, 290.68	-
CAMB3LYP MOM/aug-cc-pCVTZ	289.84, 289.87	-	290.76, 290.78	-

Nitrogen K-edge	1s(N)	
Method	Energy / eV	Intensity
CVS-EOM-IP-CCSD/aug-cc-pCVTZ	406.88	0.8866
CVS-EOM-IP-CCSD/aug-cc-pVTZ	407.17	0.8862
CVS-EOM-IP-CCSD/u-6-311(2+,+)G**	407.51	0.88405
CCSD(T) MOM/aug-cc-pCVTZ	405.78	-
CAMB3LYP MOM/aug-cc-pCVTZ	406.8	-

TABLE S2. Benchmark core-ionisation energies of pyrrole in eV at various levels of theory. The intensities are calculated as the norms of the Dyson orbitals at the EOM-IP-CCSD level.

CORE-EXCITED STATE CALCULATIONS

Carbon K-edge	$^1A_2$	$^1B_1$	$^1A_2$	$^1B_1$	$^1A_1$
Method	$1s$(C4,C5)→ π*	$1s$(C4,C5)→ π*	$1s$(C2,C3)→ π*	$1s$(C2,C3)→ π*	$1s$(C4,C5)→ σ*
CVS-ADC(2)-X/cc-pVTZ	285.91(0.0000)	285.94(0.0669)	286.64(0.0000)	286.64(0.0794)	287.44(0.0200)
CVS-EOM-EE-CCSD/cc-pVDZ	289.32(0.0000)	289.33(0.0902)	289.91(0.0000)	289.91(0.0942)	291.27(0.0230)
CVS-EOM-EE-CCSD/cc-pVTZ	286.65(0.0000)	286.66(0.0948)	287.24(0.0000)	287.24(0.0981)	288.41(0.0203)
CVS-EOM-EE-CCSD/aug-cc-pVTZ	286.50(0.0000)	286.51(0.0941)	287.07(0.0000)	287.10(0.0984)	287.41(0.0069)
CVS-EOM-EE-CCSD(fT)/u-6-311(2+,+)G**	286.99(0.0000)	287.01(0.0926)	287.63(0.0000)	287.63(0.0997)	287.66(0.0070)
SRC-R1/aug-cc-pVTZ	286.39(0.0000)	286.41(0.1220)	286.84(0.0000)	286.85(0.1332)	287.54(0.0052)
RASPT2(18/13)/cc-pVTZ	285.70(0.0000)	285.72(0.0816)	287.44(0.0000)	287.44(0.0868)	
RASPT2(18/13)/aug-cc-pVTZ	284.84(0.0000)	284.84(0.0843)	285.38(0.0000)	285.40(0.08382)	
RASPT2(24/15)/ANO-L-VTZP C_{2s}	284.98(0.0004)	284.98(0.0496)	285.25(0.0028)	285.42(0.0401)	
RASPT2(24/15)/ANO-L-VTZP C_{2v}	285.99(0.0000)	285.99(0.0822)	286.24(0.0000)	286.24(0.0707)	

Nitrogen K-edge	$^1A_1$	$^1B_1$
Method	$1s$(N)→ σ*	$1s$(N)→ π*
CVS-ADC(2)-X/cc-pVTZ	402.14(0.0127)	401.84(0.0271)
CVS-EOM-EE-CCSD/cc-pVDZ	406.56(0.0187)	406.26(0.0323)
CVS-EOM-EE-CCSD/cc-pVTZ	403.52(0.0168)	403.31(0.0336)
CVS-EOM-EE-CCSD/aug-cc-pVTZ	403.03(0.0113)	403.17(0.0339)
CVS-EOM-EE-CCSD(fT)/u-6-311(2+,+)G**	403.40(0.0112)	403.69(0.0341)
SRC1-R1/aug-cc-pVTZ	403.39(0.0186)	402.79(0.0445)
RASPT2(16/12)/cc-pVTZ	405.86(0.0117)	406.33(0.0273)
RASPT2(16/12)/aug-cc-pVTZ	403.05(0.0071)	403.19(0.0302)
RASPT2(22/15)/ANO-L-VTZP C_{2s}	402.09(0.0071)	402.61(0.0295)
RASPT2(20/14)/ANO-L-VTZP C_{2s}	403.37(0.0047)	402.20(0.0328)

TABLE S3. Calculated benchmark excitation energies in eV at the nitrogen and carbon K-edges of pyrrole at various levels of theory for the lowest-energy excited states. The oscillator strengths are provided in parenthesis for each transition.

THE LVC MODEL FOR VIBRATIONALLY RESOLVED XAS AND RAES

The highest kinetic energy features ($1h$ final states) in the resonant Auger electron spectrum (RAES) of pyrrole at the carbon K-edge exhibit a well-resolved vibrational envelope. This structure evolves with excitation energy and differs notably from the vibrational progression seen in direct valence ionisation leading to the D_0 state. The key difference arises because the final state is accessed via a resonant intermediate state, whose vibrational wave function and spectral character vary with photon energy.

To model this energy-dependent RAES within a time-independent framework, we adopt the Franck–Condon interference approach introduced by Neeb et al. [1], which was later shown by Pahl and Cederbaum [2] to be equivalent to a time-dependent formulation. In this picture, the vibrational progressions are described by the Franck–Condon factors (FCFs). If the intermediate states are long-lived, the process can be viewed in terms of a two-step mechanism: initially the excitation populates vibrational levels in the intermediate state (first set of FCFs), then Auger decay populates the final state via a second set of FCFs. However, if the intermediate state is short-lived and its lifetime is comparable to a vibrational period, then this model is no-longer valid and the intermediate state must be described as a coherent superposition of vibrational levels. This can give rise to constructive or destructive lifetime-vibrational interference among different decay pathways.

This distinction is crucial when comparing absorption- and emission-type spectra. In absorption spectra, where the short-lived state is the final state, the signal is an incoherent sum of broadened lines corresponding to individual vibrational levels. In emission spectra (Auger decay), where the short-lived state is the initial state, coherence between vibrational levels can lead to interference effects in the final state. Such coherence requires that the core-hole lifetime broadening (Γ) to be comparable to the vibrational level spacing (Table S5. For carbon $1s$ core holes ($\Gamma \approx 0.08$ eV), this condition is not met and the interference effects do not profoundly alter the spectra.

For broad excitation bandwidths, the RAES can be analysed using standard interference theory, which provides an expression for the electron emission intensity as a function of kinetic energy:

$$I(E_{\mathrm{emi}}) \propto \sum_f \left| \sum_n \frac{\langle f|n\rangle\langle n|0\rangle}{E_{\mathrm{exc}} - E_n + \frac{i\Gamma}{2}} \right|^2 \quad (1)$$

$|0\rangle$ denotes the vibrational eigenfunctions of the electronic ground state, $|n\rangle$ and E_n correspond to vibrational eigenfunctions and energies of the intermediate state, and $|f\rangle$, E_f are the final state vibrational wave functions and energies. Γ is the lifetime broadening of the core-excited state

In our experiment, however, we use synchrotron radiation, dispersed by a high-resolution monochromator, capable of providing a photon beam with a narrow bandwidth. This means that, dependent on the incoming photon energy, various specific vibrational levels in the intermediate states can be reached and they overlap differently with final states. The relation between the emission and excitation energies can be written as:

$$E_{\text{emi}} = (E_{\text{exc}} - (E_f - E_0)), \tag{2}$$

where E_0 is the vibrational energy of the ground state. If we combine the equations, the population probability of the final state is given as:

$$I(E_{\text{emi}}) \propto M_{\text{exc}} \sum_f \left| \sum_n \frac{\langle f|n\rangle\langle n|0\rangle}{E_{\text{exc}} - E_n + \frac{i\Gamma}{2}} \right|^2 \delta(E_{\text{exc}} - (E_f - E_0)) \tag{3}$$

where M_{exc} in the original paper by Neeb et al [1] introduces the monochromator function. In our case we used individual weights of the intermediate vibrational states in the XAS spectra at the given photon energy.

In our model we calculated 1D potential energy scans along vibrational coordinates for the ground electronic state, core-excited and ionised states. All states were thus described on an equal footing at the SRC1-R1/aug-cc-pVTZ level which is accurate and computationally tractable, see Figures S3 and S2. The 1D scans were performed as a function of the dimensionless normal coordinates δq_i. The particular molecular distortions were constructed to correspond to variation of the normal coordinates from $-l_i\delta q_i$ to $+r_i\delta q_i$, where r_i and l_i specify the number of steps in positive and negative directions along each normal mode. The dimensionless normal coordinate and the vector of atomic Cartesian displacements δX are defined as:

$$\delta q_i = \left(\frac{\omega_i}{\hbar}\right)^{1/2} \sum_{k=1}^{3N} L_{ki} \delta X_k \sqrt{\mathbf{M}_k}, \tag{4}$$

where L_{ki} is the orthogonal matrix obtained upon numerical diagonalization of the mass-weighted Hessian matrix and $\mathbf{M}$ is the vector of atomic masses. The Hessian matrix was

obtained for the optimized geometry of pyrrole in the ground electronic state.

First, we calculated the vibrationally-resolved XAS spectrum based on Franck–Condon factors (FCFs) between initial and intermediate core-excited states. This calculation is complicated by the fact that pyrrole possess multicentre delocalized core orbitals in the Franck–Condon point resulting in the degenerate core-excited states. The two inequivalent carbon centres give rise to symmetric and antisymmetric linear combinations of the atomic $1s$ orbitals and, as a consequence, there exist two pairs of degenerate lowest-energy core-excited states of A_2 and B_1 character. The states of A_2 character are optically dark in the Franck–Condon point. However, there exist nuclear modes which couple the A_2 and B_1 electronic states. As can be seen from the 1D potential energy scans, the most prominent vibrational modes are ν_4, ν_8, ν_{11}, ν_{12}, ν_{17} and ν_{18} for the lower energy A_2 and B_1 states corresponding to excitation from $1s$(C4, C5) and ν_4, ν_8, ν_{10}, ν_{11}, ν_{15}, ν_{18} and ν_{19} for the higher energy A_2 and B_1 states corresponding to excitation from $1s$(C2, C3). These vibrational modes were included in the linear vibronic coupling (LVC) model. Note that when performing the 1D potential energy scans at the SRC1-R1 level, the transition dipole moment of the core-excited states was dependent on the coordinates, e.g. the optically dark state in the Franck–Condon region could gain intensity. We therefore included in the model all vibrations which had a non-zero transition dipole moment along the vibrational coordinates, this applies mainly to the a_1 vibrations ν_{10} and ν_{18}. To compare the model to direct ionisation, we also included modes which were prominent in the D_0 state if accessed directly. These modes were v_6, v_7, v_{13}, v_{14}, and v_{16}. Please note that along these modes, only one core-excited states is optically bright. Table S4 provides an overview of the respective vibrational modes.

All vibrations were uncoupled, and each normal mode was considered separately. For the modes mentioned in the previous paragraph, in which the core-excited states are coupled, we used the LVC model in the diabatic representation. The starting point was the set of potential energy surfaces for the core-excited electronic states along a chosen nuclear coordinate Q. These surfaces were fitted with polynomial functions to provide smooth diabatic potential curves $V_1(Q)$ and $V_2(Q)$. The nuclear kinetic energy operator was discretized using a finite-difference approach on a uniform grid of N points spanning the relevant range of Q. The diabatic Hamiltonian for the uncoupled electronic states was constructed by adding the kinetic energy and diabatic potential matrices, forming block diagonal matrices $T + V_1$ and $T + V_2$. To incorporate vibronic coupling, a linear coupling term λq was introduced as

off-diagonal blocks connecting the two diabatic states. This results in the total Hamiltonian:

$$H = \begin{pmatrix} T + V_1 & \lambda q \\ \lambda q & T + V_2 \end{pmatrix},$$

where λ is the vibronic coupling strength parameter estimated by fitting the adiabatic energy gap between the two excited states as a function of the nuclear coordinate. The adiabatic gap was calculated as $\Delta E(q) = |E_{S_2}(q) - E_{S_1}(q)|$ using the fitted adiabatic potential energy curves. We then fitted this gap to the standard form derived from a two-level linear vibronic coupling model, $\Delta E(q) = 2\sqrt{(0.5\kappa q)^2 + (\lambda q)^2}$, where κ is the difference in the curvature of the diabatic potentials and λ represents the off-diagonal vibronic coupling. A non-linear least-squares fit yields the best-fit value of λ used in the construction of the full LVC Hamiltonian. The values of λ for ν_4, ν_8, ν_{11}, ν_{12}, ν_{17} and ν_{18} for the lower energy A_2 and B_1 states corresponding to excitation from $1s$(C4,C5) were 0.019, 0.0335, 0.0272, 0.0309, 0.0388 and 0.0356 and for ν_4, ν_8, ν_{10}, ν_{11}, ν_{15}, ν_{18} and ν_{19} for the higher energy A_2 and B_1 states corresponding to excitation from $1s$(C2,C3) were 0.0218, 0.0307, 0.0449, 0.0111, 0.0363, 0.0295, 0.0010 eV. The Hamiltonian was diagonalized to obtain coupled vibronic eigenstates and the corresponding energies, and the resulting vibronic wave functions were normalized over the nuclear coordinate grid. Transition intensities were obtained as the squared overlap integrals between the ground state and each intermediate core-excited vibronic state, and the resulting stick spectrum was plotted as a function of the energy difference between the corresponding eigenvalues. Separate spectra were constructed for the two pairs of diabatic states ($1s$(C4, C5) and $1s$(C2, C3)) which can be interpreted as contributions from individual electronic channels. Table S5 shows the assignment of the peaks. It is important to mention, that due to the core-hole lifetime, we Gaussian broadened each vibrational state by 0.08 eV, e.g. the width is significantly broader than in the case of the valence ionised states D_0 or D_1.

In the second step, we calculated vibrationally-resolved Auger spectra between intermediate core-excited states and the $1h$ participator final state (corresponding to HOMO^{-1}) for the photon energies probed in the experiment (286.04, 286.15, 286.31, 286.47, 286.58, 286.72, and 286.85 eV) As was mentioned before, since the photon bandwidth is narrower than the width defined by the core-hole lifetime, several vibrational wave functions can be excited by one photon energy. For each photon energy, we therefore constructed the overlap

between the intermediate core-excited state and the final state as an overlap between a final state and a linear combination of multidimensional FCFs in the intermediate state:

$$\sum_k c_k \prod_{i=1}^{n} \langle n_{i,k}(Q_i) \mid 0_{i,k}(Q_i)\rangle,$$

where $\prod_{i=1}^{n}\langle n_i(Q_i) \mid 0_i(Q_i)\rangle$ is the individual FCF weighted by c_k which is calculated from the contribution of each multidimensional FCF to the intensity in the XAS spectrum at a given photon energy. The overall spectrum across all photon energies was constructed again for the two pairs of diabatic states separately ($1s$(C4,C5) and $1s$(C2,C3)), which can be interpreted as contributions from individual electronic channels.

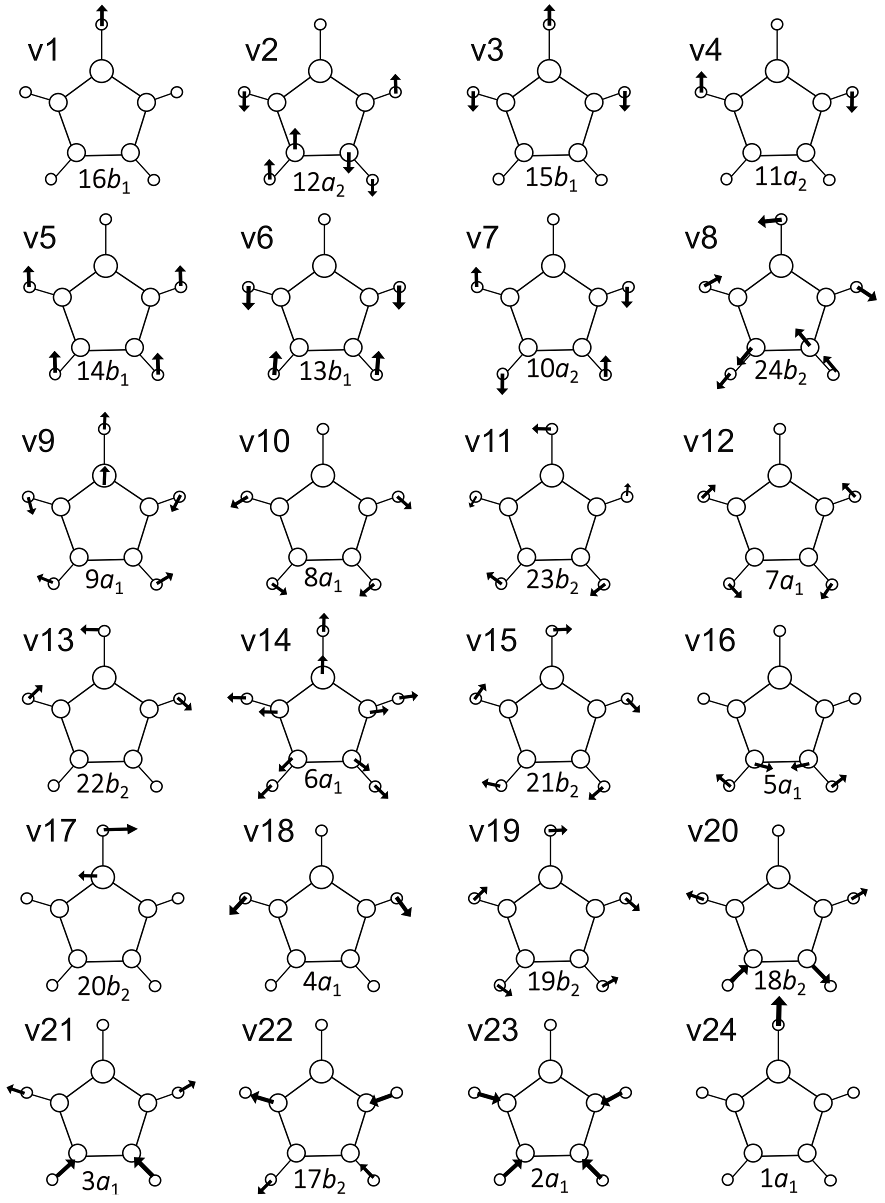

FIG. S2. Computed forms of the vibrational modes of pyrrole in the electronic ground state at the SRC1-R1/aug-cc-pVTZ level. The Herzberg labels were adapted from Ref. [3].

	Analytical		Fitted	Fitted				
Number	Herzberg Labels	Ground State	Ground State	D_0	S_1	S_2	S_3	S_4
ν_4	$11a_2$	0.086	0.085	0.085	0.092*	0.092*	0.096*	0.083*
ν_6	$13b_1$	0.104	0.084	0.097	0.064	0.068	0.072	0.073
ν_7	$10a_2$	0.108	0.097	0.102	0.064	0.069	0.070	0.071
ν_8	$24b_2$	0.109	0.110	0.112	0.119*	0.118*	0.122*	0.121*
ν_{10}	$8a_1$	0.127	0.130	0.130	0.135	0.138	0.130*	0.134*
ν_{11}	$23b_2$	0.132	0.134	0.134	0.141*	0.139*	0.132*	0.139*
ν_{12}	$7a_1$	0.134	0.134	0.134	0.135*	0.139*	0.138	0.145
ν_{13}	$22b_2$	0.143	0.145	0.146	0.145	0.152	0.135	0.139
ν_{14}	$6a_1$	0.144	0.149	0.172	0.153	0.154	0.160	0.171
ν_{15}	$21b_2$	0.163	0.164	0.165	0.183	0.187	0.198*	0.189*
ν_{16}	$5a_1$	0.174	0.173	0.184	0.190	0.197	0.178	0.191
ν_{17}	$20b_2$	0.179	0.180	0.180	0.200*	0.197*	0.194	0.208
ν_{18}	$4a_1$	0.185	0.185	0.184	0.212*	0.212*	0.212*	0.207*
ν_{19}	$19b_2$	0.194	0.195	0.197	0.194	0.204	0.210*	0.216*

TABLE S4. Vibrational modes relevant for the description of the XAS, the valence photoelectron spectrum and the RAES at the carbon K-edge. The table contains analytical vibrational frequencies in eV (in the column 'analytical') for the ground electronic state, and frequencies in eV obtained from the fitted potential energy surfaces for the ground electronic state, the ionised state D_0 and the core-excited states. The asterisk shows vibrational frequencies of the diabatic states. The Herzberg labels were adapted from Ref. [3].

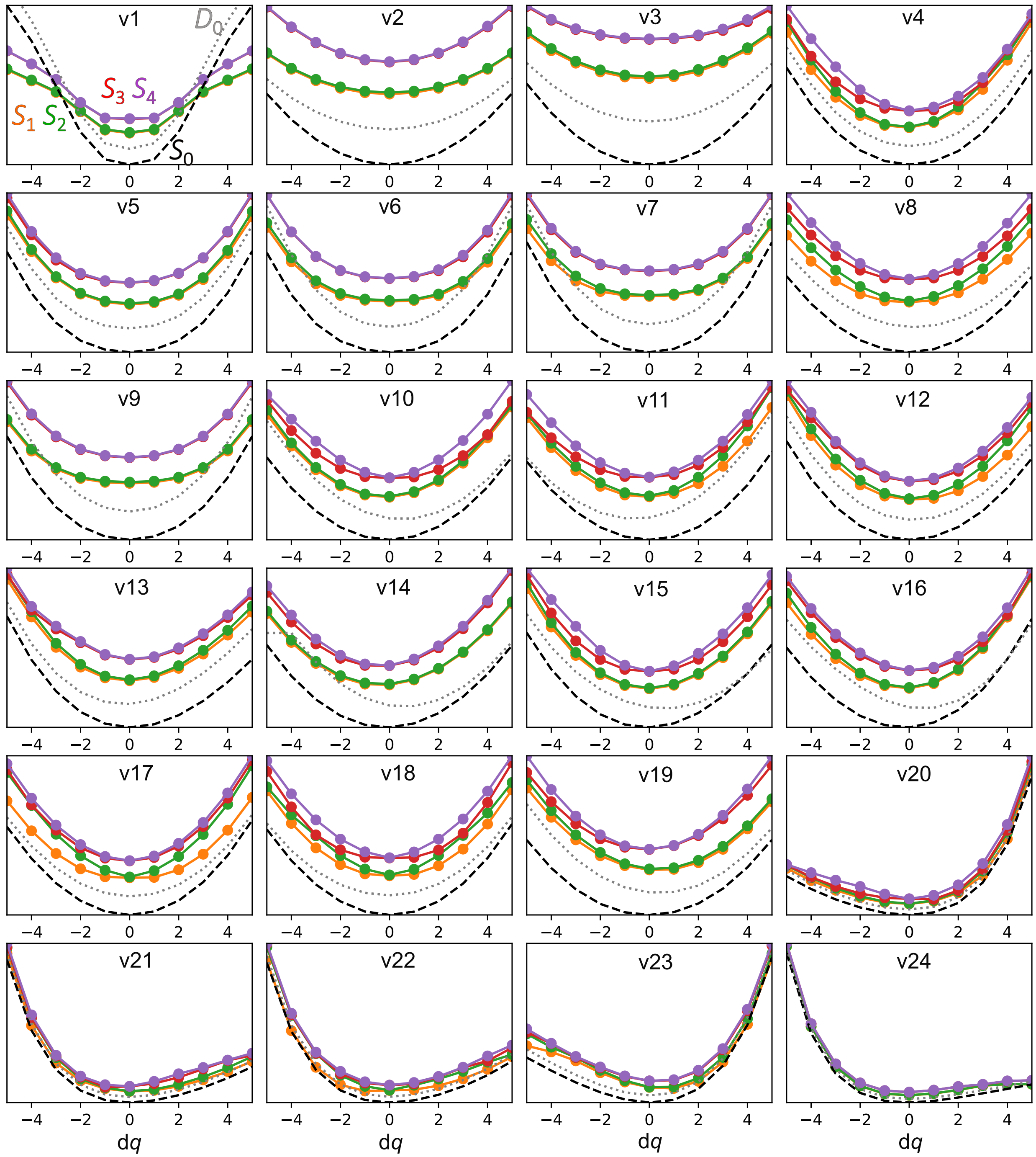

FIG. S3. Potential energy profiles along the 24 vibrational modes of pyrrole calculated at the SRC1-R1/aug-cc-pVTZ level; the energy levels are shifted on the energy axis to fit the figure. The gray dotted line corresponds to the D_0 ionised state, the black dashed line to the ground electronic state S_0, and the color lines represent four lowest-energy core-excited states S_1 (orange), S_2 (green), S_3 (red), S_4 (purple). dq is a dimensionless coordinate.

$S_0 \rightarrow S_1, S_2, S_3, S_4$			
Energy / eV	Intensity	Vibrational Transition	Herzberg Notation
286.082	0.16902	S_1(0)	0
286.174	0.03348	S_1(1v4)	$11a_2$
286.201	0.01680	S_1(1v8)	$24b_2$
286.217	0.03462	S_1(1v12)	$7a_1$
286.223	0.02339	S_1(1v11)	$22b_2$
286.266	0.00378	S_1(2v4)	$11a_2$
286.282	0.04028	S_1(1v17)	$20b_2$
286.294	0.07085	S_1(1v18)	$4a_1$
286.386	0.01403	S_1(1v4,1v18)	$11a_2$,$4a_1$
286.415	0.00704	S_1(1v8,1v18)	$24b_2$,$4a_1$
286.420	0.00825	S_1(1v12,1v17)	$7a_1$,$20b_2$
286.436	0.01451	S_1(1v11,1v18)	$23b_2$,$4a_1$
286.437	0.00980	S_1(1v12,1v18)	$7a_1$,$4a_1$
286.494	0.01688	S_1(1v17,1v18)	$20b_2$,$4a_1$
286.142	0.27057	S_2(0)	0
286.261	0.01701	S_2(1v8)	$24b_2$
286.282	0.00801	S_2(1v11)	$22b_2$
286.282	0.01129	S_2(1v12)	$7a_1$
286.340	0.04733	S_2(1v17)	$20b_2$
286.355	0.08861	S_2(1v18)	$4a_1$
286.480	0.00197	S_2(1v11,1v12)	$23b_2$,$7a_1$
286.553	0.01550	S_2(1v17,1v18)	$20b_2$,$4a_1$
286.523	0.17928	S_3(0)	0
286.619	0.07441	S_3(1v4)	$11a_2$
286.645	0.02133	S_3(1v8)	$24b_2$
286.653	0.04411	S_3(1v10)	$8a_1$
286.655	0.01754	S_3(1v11)	$23b_2$
286.712	0.00183	S_3(1v15)	$21b_2$
286.735	0.10472	S_3(1v18)	$4a_1$
286.733	0.00150	S_3(1v19)	$19b_2$
286.749	0.01831	S_3(1v4,1v10)	$11a_2$,$8a_1$
286.751	0.00728	S_3(1v4,1v11)	$11a_2$,$23b_2$
286.857	0.01246	S_3(1v8,1v18)	$24b_2$,$4a_1$
286.865	0.02576	S_3(1v10,1v18)	$8a_1$,$4a_1$
286.867	0.01025	S_3(1v11,1v18)	$23b_2$,$4a_1$
286.596	0.23010	S_4(0)	0
286.717	0.02610	S_4(1v8)	$24b_2$
286.730	0.04856	S_4(1v10)	$8a_1$
286.735	0.00286	S_4(1v11)	$23b_2$
286.794	0.01176	S_4(1v15)	$21b_2$
286.803	0.13109	S_4(1v18)	$4a_1$
286.851	0.00550	S_4(1v8,1v10)	$24b_2$,$8a_1$
286.924	0.01487	S_4(1v8,1v18)	$24b_2$,$4a_1$
286.937	0.02766	S_4(1v10,1v18)	$8a_1$,$4a_1$
287.001	0.00670	S_4(1v15,1v18)	$21b_2$,$4a_1$

TABLE S5. Vibrationally resolved XAS spectrum of pyrrole. The core-excited states are named S_1, S_2 for the C4,C5 carbon atom sites and S_3, S_4 for the C2,C3 carbon sites. The electronic states were calculated at the SRC1-R1/aug-cc-pVTZ level, and for the construction of the spectrum the parallel normal modes approximation was employed. The vibrational state assignment is provided as state(number of quanta—v—normal mode's number). Intensities were normalized to 1.

RESONANT AUGER SPECTRA

nitrogen K-edge			
Core Excited $^1A_1$ ($1s$(N)$\rightarrow$ σ*)			
Energy / eV	Width/10^4 a.u.	Transition	
389.92	0.00610	$9a_1$	participator
388.25	0.57045	$8a_1$	participator
384.90	0.21919	$7a_1$	participator
383.61	0.43699	$6a_1$	participator
381.18	0.00067	$1a_2$,$1a_2\rightarrow$ $10a_1$	spectator
380.41	0.00092	$1a_2$,$1a_2\rightarrow$ $13a_1$	spectator
394.62	0.00139	$1a_2$	participator
389.38	0.04849	$6b_2$	participator
388.46	0.49793	$5b_2$	participator
384.35	0.28421	$4b_2$	participator
381.40	0.00000		spectator
393.84	0.62552	$2b_1$	participator
389.19	0.59034	$1b_1$	participator
Core Excited $^1B_1$ ($1s$(N)$\rightarrow$ π^*)			
Energy / eV	Width/10^4 a.u.	Transition	
390.06	0.02030	$9a_1$	participator
388.39	0.29699	$8a_1$	participator
385.03	0.15138	$7a_1$	participator
383.75	0.29802	$6a_1$	participator
381.32	0.00014	$1a_2$,$1a_2\rightarrow$ $10a_1$	spectator
380.55	0.00012	$1a_2$,$1a_2\rightarrow$ $13a_1$	spectator
394.76	0.00534	$1a_2$	participator
389.52	0.03959	$6b_2$	participator
388.6	0.36826	$5b_2$	participator
384.49	0.19225	$4b_2$	participator
381.54	0.00000		spectator
393.98	0.56791	$2b_1$	participator
389.33	0.38797	$1b_1$	participator

TABLE S6. Auger electron energies in eV and partial decay widths in a.u. at the CVS-EOM-CCSD/aug-cc-pVTZ level at the nitrogen K-edge.

Nitrogen K-edge			
Core Excited $^1A_1$ (1s(N)→ σ*)			
Energy / eV	Width/10^4 a.u.	Transition	
390.42	0.00538	$9a_1$	participator
388.72	0.56151	$8a_1$	participator
385.37	0.20425	$7a_1$	participator
384.07	0.44497	$6a_1$	participator
381.92	0.00071	$1a_2$,$1a_2$→ $10a_1$	spectator
381.1	0.00072	$1a_2$,$1a_2$→ $19a_1$	spectator
395.17	0.00109	$1a_2$	participator
389.87	0.05104	$6b_2$	participator
388.93	0.47245	$5b_2$	participator
384.83	0.27010	$4b_2$	participator
382.17	0.00000		spectator
394.41	0.61525	$2b_1$	participator
389.77	0.56454	$1b_1$	participator
Core Excited $^1B_1$ (1s(N)→ π*)			
Energy / eV	Width/10^4 a.u.	Transition	
390.71	0.01908	$9a_1$	participator
389.01	0.30202	$8a_1$	participator
385.66	0.14821	$7a_1$	participator
384.36	0.30138	$6a_1$	participator
382.21	0.00012	$1a_2$,$1a_2$→ $10a_1$	spectator
381.39	0.00017	$1a_2$,$1a_2$→ $19a_1$	spectator
395.46	0.00473	$1a_2$	participator
390.16	0.04361	$6b_2$	participator
389.22	0.37381	$5b_2$	participator
385.12	0.19347	$4b_2$	participator
382.46	0.00000		spectator
394.7	0.59241	$2b_1$	participator
390.06	0.38818	$1b_1$	participator

TABLE S7. Auger electron energies in eV and partial decay widths in a.u. at the CVS-EOM-CCSD(fT)/u-6-311(2+,+)G** level at the nitrogen K-edge. These results are plotted in Figure 6.

Nitrogen K-edge			
Core Excited $^1A_1$ (1s(N)$\rightarrow$ σ*)			
Energy / eV	Width/10^{-4} a.u.	Transition	
390.47	0.01183	$9a_1$	participator
389.97	0.04988	$6b_2$	participator
389.09	0.96674	$5b_3$	participator
388.55	0.78588	$8a_1$	participator
386.88	0.13452		spectator
385.91	0.31232	$7a_1$	participator
384.91	0.17114		spectator
384.84	0.92853		spectator
384.63	0.63332	$4b_2$	participator
384.51	0.02546		spectator
384.29	0.05654		spectator
379.88	0.74651		spectator
395.23	0.00000	$1a_2$	participator
394.42	1.49498	$2b_1$	participator
390.62	0.90398	$1b_1$	participator
389.11	0.09525		spectator
381.62	0.05138		spectator
380.84	0.38645		spectator
380.75	0.07682		spectator
380.65	0.34844		spectator
380.55	0.19142		spectator
380.19	0.13267		spectator

TABLE S8. Auger electron energies in eV and partial decay widths in a.u. at the RASPT2/ANO-L-TZVP level at the nitrogen K-edge excitation (1s(N)$\rightarrow$ σ* state) forming the $^1A_1$ state. The table provides participator channels and spectators channels with partial decay widths higher than 0.5 $\times 10^{-5}$ a.u. These results are plotted in Figure S4.

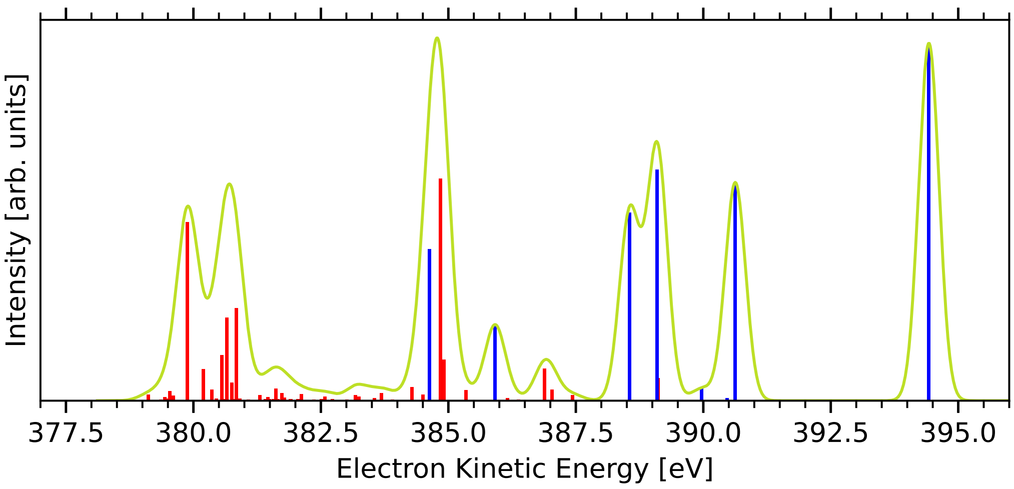

FIG. S4. Auger electron spectrum calculated at the RASPT2/ANO-L-TZVP level at the nitrogen K-edge excitation (1s(N)→ σ* state) forming the $^1A_1$ state (see Table S8). The peaks were Gaussian broadened by a phenomenological 0.2 eV. Participator channels are visualized as blue sticks and spectators channels as red sticks.

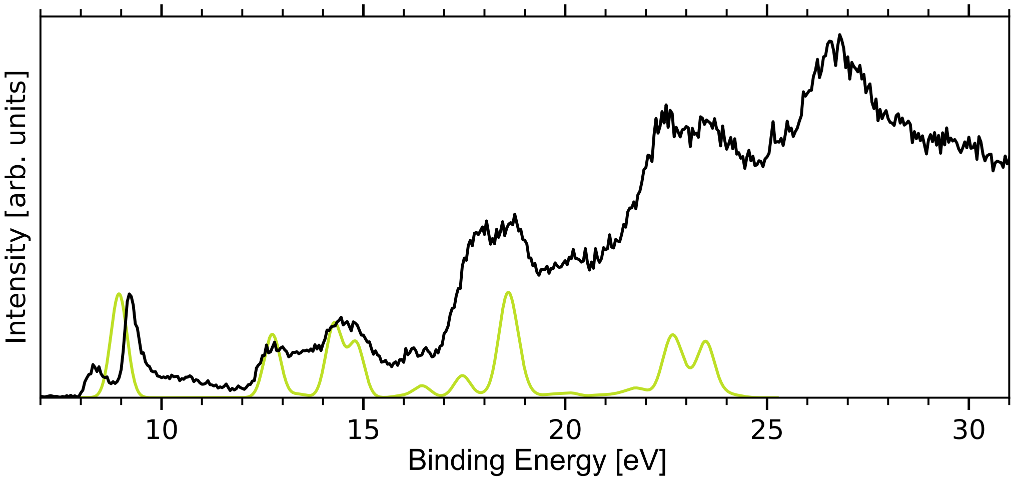

FIG. S5. Comparison of the experimental Auger electron spectrum, recorded at a photon energy of 402.9 eV, plotted in binding energy (black) and the spectrum calculated at the RASPT2/ANO-L-TZVP level at the nitrogen K-edge excitation (1s(N)→ σ* state) forming the $^1A_1$ state (see Table S8) (green). The theoretical peaks were Gaussian broadened by a phenomenological 0.2 eV and normalized with respect to the lowest-energy peak. The theoretical spectrum has not been shifted.

Nitrogen K-edge			
Core Excited $^1B_1$ ($1s$(N)$\rightarrow\pi^*$)			
Energy / eV	Width/10^{-4} a.u.	Transition	
389.29	0.00102	$9a_1$	participator
388.79	0.02547	$6b_2$	participator
387.92	0.44664	$5b_2$	participator
387.38	0.32972	$8a_1$	participator
384.74	0.07996	$7a_1$	participator
383.46	0.05669	$4b_2$	participator
382.94	0.01692		spectator
382.38	0.16433		spectator
382.15	0.12884		spectator
382.07	0.08009		spectator
382.02	0.10773		spectator
381.84	0.24031		spectator
381.33	0.23080		spectator
380.87	0.05201		spectator
380.61	0.11872		spectator
380.12	0.07606		spectator
379.96	0.32612		spectator
379.45	0.06200		spectator
378.96	0.13618		spectator
394.05	0.00009	$1a_2$	participator
393.25	0.76087	$2b_1$	participator
389.45	0.13683	$1b_1$	participator
387.94	0.05547		spectator
386.26	0.25371		spectator
384.98	0.76088	$2b_1$,$2b_1\rightarrow 2a_2$, $3b_1$	spectator

TABLE S9. Auger electron energies in eV and partial decay widths in a.u. at the RASPT2/ANO-L-TZVP level at the nitrogen K-edge excitation ($1s$(N)$\rightarrow\pi^*$ state) forming the $^1B_1$ state. The table provides participator channels and spectators channels with partial decay widths higher than 0.5 $\times 10^{-5}$ a.u. These results are plotted in Figure S6.

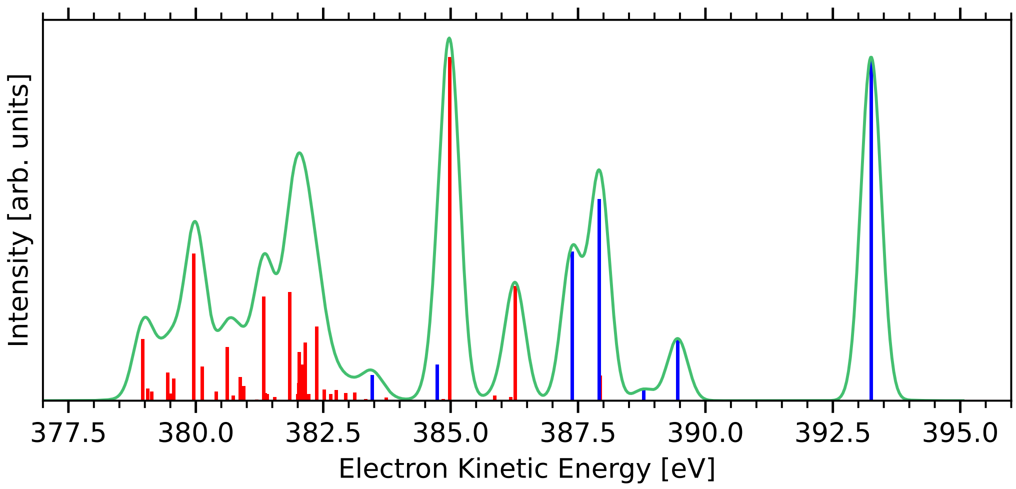

FIG. S6. Auger electron spectrum calculated at the RASPT2/ANO-L-TZVP level at the nitrogen K-edge excitation ($1s$(N)$\rightarrow$ π^* state) forming the $^1B_1$ state (see Table S9). The peaks were Gaussian broadened by a phenomenological 0.2 eV. Participator channels are visualized as blue sticks and spectators channels as red sticks.

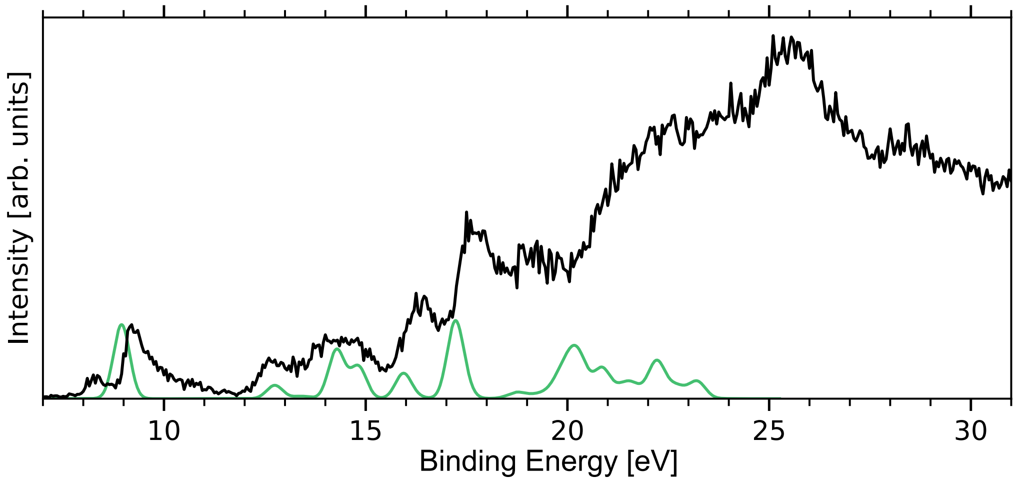

FIG. S7. Comparison of the experimental Auger electron spectrum, recorded at a photon energy of 401.9 eV, plotted in binding energy (black) and the spectrum calculated at the RASPT2/ANO-L-TZVP level at the nitrogen K-edge excitation ($1s$(N)$\rightarrow$ π^* state) forming the $^1B_1$ state (see Table S9) (green). The theoretical peaks were Gaussian broadened by a phenomenological 0.2 eV and normalized with respect to the lowest-energy peak. The theoretical spectrum has not been shifted.

Carbon K-edge			
Core Excited $^1B_1$ ($1s$(C4,C5)$\rightarrow \pi^*$)			
Energy / eV	Width/10^4 a.u.	Transition	
273.42	0.88708	$9a_1$	participator
271.75	0.47979	$8a_1$	participator
268.39	0.10640	$7a_1$	participator
267.1	0.54976	$6a_1$	participator
278.12	0.55160	$1a_2$	participator
272.88	0.37029	$6b_2$	participator
271.96	0.10098	$5b_2$	participator
267.84	1.11542	$4b_2$	participator
277.34	0.44261	$2b_1$	participator
272.69	0.12692	$1b_1$	participator
Core Excited $^1B_1$ ($1s$(C2,C3)$\rightarrow \pi^*$)			
Energy / eV	Width/10^4 a.u.	Transition	
274.01	0.33586	$9a_1$	participator
272.34	0.36496	$8a_1$	participator
268.98	1.17755	$7a_1$	participator
267.69	0.61833	$6a_1$	participator
278.71	0.30645	$1a_2$	participator
273.47	0.28202	$6b_2$	participator
272.54	0.64405	$5b_2$	participator
268.43	0.17969	$4b_2$	participator
277.93	0.05713	$2b_1$	participator
273.28	0.36949	$1b_1$	participator

TABLE S10. Auger electron energies in eV and partial decay widths in a.u. at the CVS-EOM-CCSD/aug-cc-pVTZ level at the carbon K-edge.

Carbon K-edge			
Core Excited $^1B_1$ ($1s$(C4,C5)$\rightarrow \pi^*$)			
Energy / eV	Width/10^4 a.u.	Transition	
274.04	0.90816	$9a_1$	participator
272.34	0.49442	$8a_1$	participator
268.98	0.10634	$7a_1$	participator
267.68	0.54161	$6a_1$	participator
265.57	0.00014	$1a_2$, $1a_2 \rightarrow$ $10a_1$, $19a_1$	spectator
264.75	0.01153	$1a_2$, $1a_2 \rightarrow 19a_1$, $23a_1$	spectator
278.79	0.58181	$1a_2$	participator
273.48	0.37372	$6b_2$	participator
272.54	0.09744	$5b_2$	participator
268.44	1.08832	$4b_2$	participator
265.81	0.00000	$1a_2$, $2b_1 \rightarrow$ $19a_1$, $10a_1$	spectator
278.03	0.45740	$2b_1$	participator
273.38	0.13275	$1b_1$	participator
Core Excited $^1B_1$ ($1s$(C2,C3)$\rightarrow \pi^*$)			
Energy / eV	Width/10^4 a.u.	Transition	
274.65	0.34290	$9a_1$	participator
272.95	0.38053	$8a_1$	participator
269.59	1.16505	$7a_1$	participator
268.30	0.58455	$6a_1$	participator
266.18	0.00041	$1a_2$, $1a_2 \rightarrow$ $10a_1$, $19a_1$	spectator
265.36	0.00170	$1a_2$, $1a_2 \rightarrow 19a_1$, $23a_1$	spectator
279.41	0.31733	$1a_2$	participator
274.10	0.27399	$6b_2$	participator
273.16	0.66419	$5b_2$	participator
269.06	0.17379	$4b_2$	participator
266.41	0.00000	$1a_2$, $2b_1 \rightarrow$ $19a_1$, $10a_1$	spectator
278.64	0.05684	$2b_1$	participator
274.00	0.36551	$1b_1$	participator

TABLE S11. Auger electron energies in eV and partial decay widths in a.u. at the CVS-EOM-CCSD(fT)/u-6-311(2+,+)G** level at the carbon K-edge. These results are plotted in Figure 7.

Carbon K-edge			
Core Excited $^1B_1$ ($1s$(C4,C5)$\rightarrow \pi^*$)			
Energy / eV	Width/10^{-4} a.u.	Transition	
274.31	1.1087	$9a_1$	participator
273.82	0.5850	$6b_2$	participator
272.75	0.0068	$5b_2$	participator
272.96	0.1171		spectator
272.68	0.3747	$8a_1$	participator
272.67	1.3511		spectator
272.33	0.2033		spectator
271.15	0.1304		spectator
270.83	0.6288		spectator
269.90	0.6544		spectator
269.73	0.2005		spectator
269.47	0.0425	$7a_1$	participator
269.07	1.0658		spectator
268.62	0.0007	$4b_2$	participator
268.68	0.3978		spectator
268.23	0.5849		spectator
267.95	0.2866		spectator
267.82	0.1486		spectator
267.74	0.4228	$6a_1$	participator
267.53	0.2366		spectator
267.24	0.2247		spectator
266.91	0.3453		spectator
266.76	0.1729		spectator
266.47	0.1108		spectator
266.35	0.6041		spectator
263.08	0.4011		spectator
266.34	0.1182		spectator
265.83	0.2151		spectator
264.53	0.1339		spectator
279.21	1.3620	$1a_2$	participator
278.37	1.7161	$2b_1$	participator
274.57	0.2053	$1b_1$	participator
272.46	0.9618	$1a_2$,$1a_2\rightarrow 2a_2$	spectator

TABLE S12. Auger electron energies in eV and partial decay widths in a.u. at the RASPT2/ANO-L-TZVP level at the carbon K-edge excitation ($1s$(C4,C5)$\rightarrow \pi^*$) forming the $^1B_1$ state. The table provides participator channels and spectators channels with partial decay widths higher than $\times 10^{-5}$ a.u. These results are plotted in Figure S8.

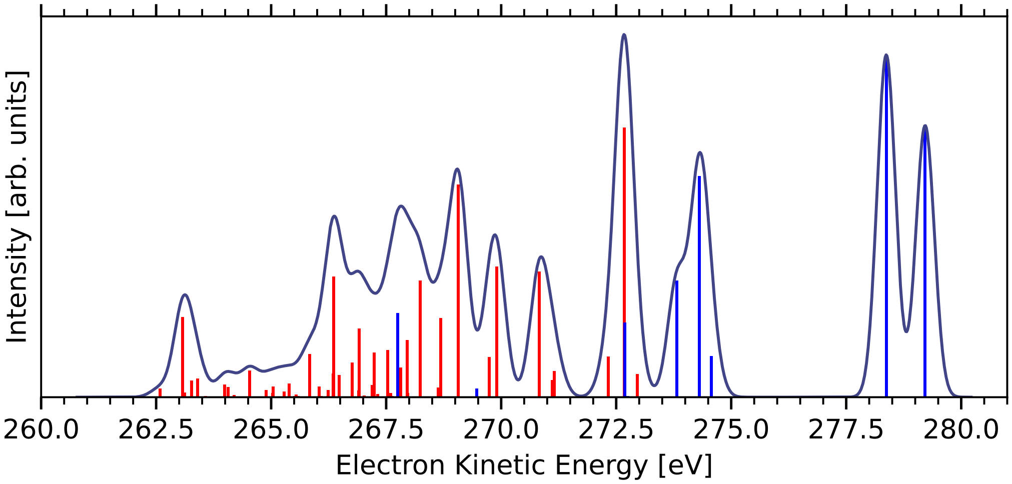

FIG. S8. Auger electron spectrum the carbon K-edge at the RASPT2/ANO-L-TZVP level at the carbon K-edge excitation (1s(C4,C5)$\rightarrow \pi^*$) forming the $^1B_1$ state (see Table S12). The peaks were Gaussian broadened by a phenomenological 0.2 eV. Participator channels are visualized as blue sticks, spectators channels as red sticks.

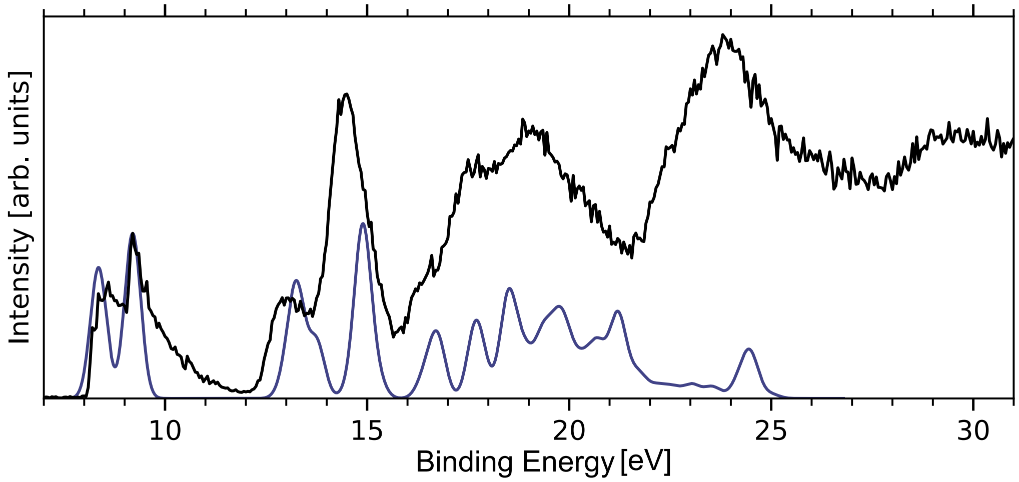

FIG. S9. Comparison of the experimental Auger electron spectrum, recorded at a photon energy of 285.9 eV, plotted in binding energy (black) and the spectrum calculated at the RASPT2/ANO-L-TZVP level at the carbon K-edge excitation (1s(C4,C5)$\rightarrow \pi^*$) forming the $^1B_1$ state (see Table S12) (blue). The theoretical peaks were Gaussian broadened by a phenomenological 0.2 eV and normalized with respect to the lowest-energy peak. The theoretical spectrum has been shifted by 2 eV.

Carbon K-edge			
Core Excited $^1B_1$ ($1s$(C2,C3)$\rightarrow \pi^*$)			
Energy / eV	Width/10^{-4} a.u.	Transition	
273.82	0.0618	$9a_1$	participator
273.32	0.0850	$6b_2$	participator
272.26	0.2162	$5b_2$	participator
272.19	0.0683	$8a_1$	participator
268.99	0.0108	$7a_1$	participator
268.58	0.1985		spectator
268.19	0.3208		spectator
268.12	0.0675	$4b_2$	participator
267.48	0.1760		spectator
267.33	0.1908		spectator
267.14	0.2109		spectator
267.24	0.0131	$6a_1$	participator
267.11	0.4183		spectator
263.55	0.1005		spectator
278.72	1.2324	$1a_2$	participator
277.89	0.0001	$2b_1$	participator
274.09	0.1193	$1b_1$	participator
272.96	0.1171		spectator
272.67	1.3511	$1a_2$,$1a_2\rightarrow 2a_2$	spectator
272.32	0.2033		spectator
271.15	0.1304		spectator

TABLE S13. Auger electron energies in eV and partial decay widths in a.u. at the RASPT2/ANO-L-TZVP level at the carbon K-edge excitation ($1s$(C2,C3)$\rightarrow \pi^*$) forming the $^1B_1$ state. The table provides participator channels and spectators channels with partial decay widths higher than $\times 10^{-5}$ a.u. These results are plotted in Figure S10.

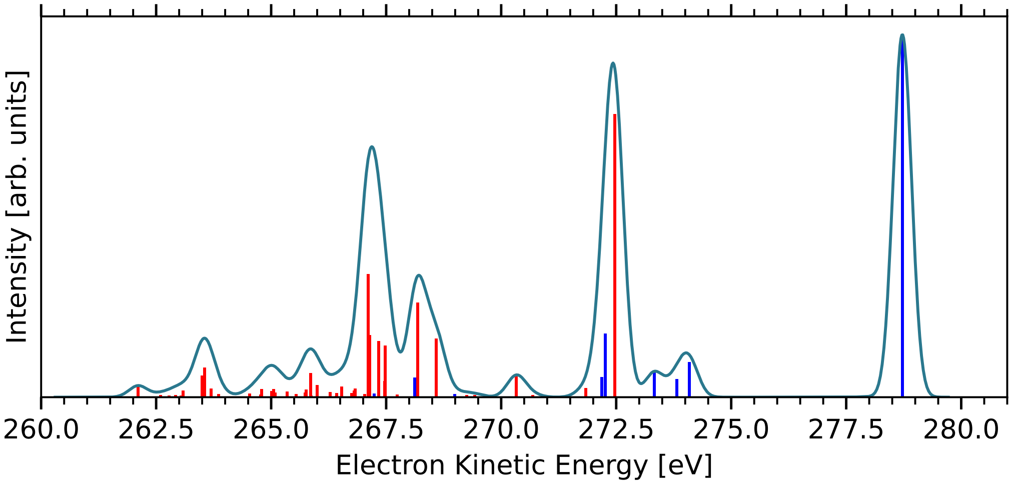

FIG. S10. Auger electron spectrum the carbon K-edge at the RASPT2/ANO-L-TZVP level at the carbon K-edge excitation ($1s$(C2,C3)$\rightarrow \pi^*$) forming the $^1B_1$ state (see Table S13). The peaks were Gaussian broadened by a phenomenological 0.2 eV. Participator channels are visualized as blue sticks, spectators channels as red sticks.

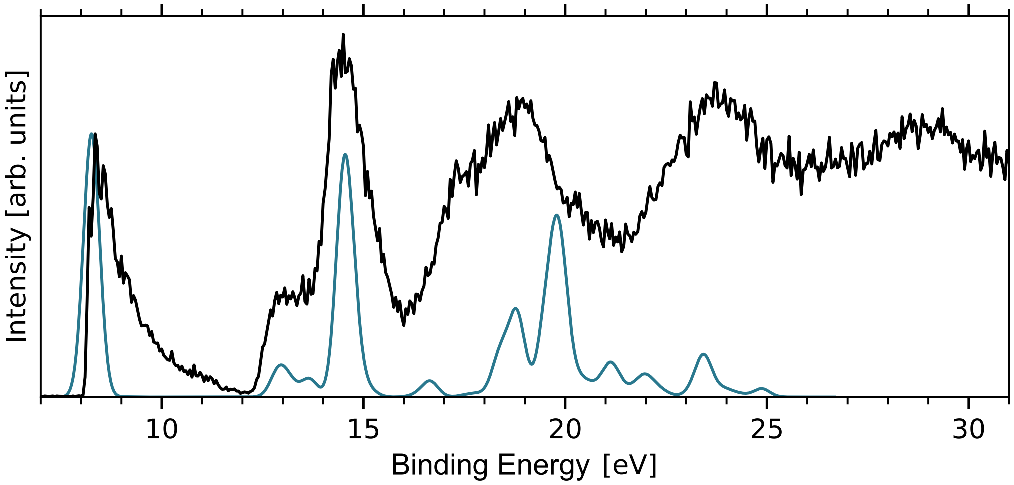

FIG. S11. Comparison of the experimental Auger electron spectrum, recorded at a photon energy of 286.5 eV, plotted in binding energy (black) and the spectrum calculated at the RASPT2/ANO-L-TZVP level at the carbon K-edge excitation ($1s$(C2,C3)$\rightarrow \pi^*$) forming the $^1B_1$ state (see Table S13) (blue). The theoretical peaks were Gaussian broadened by a phenomenological 0.2 eV and normalized with respect to the lowest-energy peak. The theoretical spectrum has been shifted by 2 eV.

VIBRATIONALLY RESOLVED RESONANT AUGER SPECTRA

$S_1 \rightarrow D_0$			
Energy / eV	Intensity	Vibrational Transition	Herzberg Notation
8.200	1.00000	$D_0(0)$	0
8.285	0.33560	$D_0(1v4)$	$11a_2$
8.311	0.17627	$D_0(1v8)$	$24b_2$
8.330	0.25799	$D_0(1v10)$	$8a_1$
8.334	0.11553	$D_0(1v12)$	$7a_1$
8.365	0.18774	$D_0(1v15)$	$21b_2$
8.371	0.22110	$D_0(1v14)$	$6a_1$
8.384	0.23293	$D_0(1v16)$	$5a_1$
8.380	0.41710	$D_0(1v17)$	$20b_2$
8.384	0.71871	$D_0(1v18)$	$4a_1$
8.469	0.07817	$D_0(1v4,1v16)$	$11a_2$,$5a_1$
8.465	0.13998	$D_0(1v4,1v17)$	$11a_2$,$20b_2$
8.469	0.24121	$D_0(1v4,1v18)$	$11a_2$,$4a_1$
8.495	0.12669	$D_0(1v8,1v18)$	$24b_2$,$4a_1$
8.510	0.10761	$D_0(1v10,1v17)$	$8a_1$,$20b_2$
8.514	0.18542	$D_0(1v10,1v18)$	$8a_1$,$4a_1$
8.545	0.07831	$D_0(1v15,1v17)$	$21b_2$,$20b_2$
8.549	0.13494	$D_0(1v15,1v18)$	$21b_2$,$4a_1$
8.551	0.09223	$D_0(1v14,1v17)$	$6a_1$,$20b_2$
8.555	0.15891	$D_0(1v14,1v18)$	$6a_1$,$4a_1$
8.564	0.09716	$D_0(1v16,1v17)$	$5a_1$,$20b_2$
8.564	0.29977	$D_0(1v17,1v18)$	$20b_2$,$4a_1$
8.568	0.16742	$D_0(1v16,1v18)$	$5a_1$,$4a_1$

TABLE S14. Vibrational progressions in the RAES due to deexcitation from the intermediate core-excited diabatic state S_1 to the final ionised state D_0 calculated at the SRC1-R1/aug-cc-pVTZ level using the parallel normal modes approximation. The vibrational state assignment is provided as state(number of quanta—v—normal mode's number). Intensities were normalized to 1.

$S_2 \rightarrow D_0$			
Energy / eV	Intensity	Vibrational Transition	Herzberg Notation
8.200	1.00000	$D_0(0)$	0
8.312	0.10108	D_0(1v8)	$24b_2$
8.330	0.25800	D_0(1v10)	$8a_1$
8.334	0.41968	D_0(1v11)	$23b_2$
8.334	0.16469	D_0(1v12)	$7a_1$
8.347	0.01255	D_0(1v13)	$22b_2$
8.371	0.22110	D_0(1v14)	$6a_1$
8.365	0.18774	D_0(1v15)	$21b_2$
8.384	0.23294	D_0(1v16)	$5a_1$
8.381	0.51052	D_0(1v17)	$20b_2$
8.384	0.60273	D_0(1v18)	$4a_1$
8.464	0.10827	D_0(1v10,1v11)	$8a_1$,$23b_2$
8.511	0.13171	D_0(1v10,1v17)	$8a_1$,$20b_2$
8.514	0.15550	D_0(1v10,1v18)	$8a_1$,$4a_1$
8.515	0.08407	D_0(1v12,1v17)	$7a_1$,$20b_2$
8.515	0.21425	D_0(1v11,1v17)	$23b_2$,$20b_2$
8.518	0.09927	D_0(1v12,1v18)	$7a_1$,$4a_1$
8.518	0.25295	D_0(1v11,1v18)	$23b_2$,$4a_1$
8.518	0.09776	D_0(1v16,1v11)	$5a_1$,$23b_2$
8.539	0.05076	D_0(2v14)	$6a_1$
8.546	0.09585	D_0(1v15,1v17)	$21b_2$,$20b_2$
8.549	0.11316	D_0(1v15,1v18)	$21b_2$,$4a_1$
8.552	0.11288	D_0(1v14,1v17)	$6a_1$,$20b_2$
8.555	0.13326	D_0(1v14,1v18)	$6a_1$,$4a_1$

TABLE S15. Vibrational progressions in the RAES due to deexcitation from the intermediate core-excited diabatic state S_2 to the final ionised state D_0 calculated at the SRC1-R1/aug-cc-pVTZ level using the parallel normal modes approximation. The vibrational state assignment is provided as state(number of quanta—v—normal mode's number). Intensities were normalized to 1.

$S_3 \rightarrow D_0$			
Energy / eV	Intensity	Vibrational Transition	Herzberg notation
8.200	1.00000	D_0(0)	0
8.286	0.60223	D_0(1v4)	$11a_2$
8.312	0.10740	D_0(1v8)	$24b_2$
8.330	0.10662	D_0(1v10)	$8a_1$
8.347	0.11206	D_0(1v13)	$22b_2$
8.365	0.16424	D_0(1v15)	$21b_2$
8.371	0.06054	D_0(2v4)	$11a_2$
8.372	0.26407	D_0(1v14)	$6a_1$
8.384	1.00880	D_0(1v18)	$4a_1$
8.385	0.08828	D_0(1v16)	$5a_1$
8.397	0.13561	D_0(1v19)	$19b_2$
8.451	0.09892	D_0(1v4,1v15)	$11a_2$,$21b_2$
8.458	0.15903	D_0(1v14,1v4)	$6a_1$,$11a_2$
8.460	0.02368	D_0(2v10)	$8a_1$
8.470	0.60753	D_0(1v4,1v18)	$11a_2$,$4a_1$
8.483	0.08167	D_0(1v4,1v19)	$11a_2$,$19b_2$
8.496	0.10834	D_0(1v8,1v18)	$24b_2$,$4a_1$
8.514	0.10756	D_0(1v10,1v18)	$8a_1$,$4a_1$
8.531	0.11305	D_0(1v13,1v18)	$22b_2$,$4a_1$
8.549	0.16569	D_0(1v15,1v18)	$21b_2$,$4a_1$
8.556	0.26640	D_0(1v14,1v18)	$6a_1$,$4a_1$
8.569	0.08906	D_0(1v16,1v18)	$5a_1$,$4a_1$
8.635	0.09978	D_0(1v4,1v15,1v18)	$11a_2$,$21b_2$,$4a_1$
8.642	0.16043	D_0(1v14,1v4,1v18)	$6a_1$,$11a_2$,$4a_1$

TABLE S16. Vibrational progressions in the RAES due to deexcitation from the intermediate core-excited state S_3 to the final ionised state D_0 calculated at the SRC1-R1/aug-cc-pVTZ level using the parallel normal modes approximation. The vibrational state assignment is provided as state(number of quanta—v—normal mode's number). Intensities were normalized to 1.

$S_4 \rightarrow D_0$			
Energy / eV	Intensity	Vibrational Transition	Herzberg notation
8.200	1.00000	$D_0(0)$	0
8.285	0.36975	D_0(1v4)	$11a_2$
8.311	0.12795	D_0(1v8)	$24b_2$
8.330	0.09549	D_0(1v10)	$8a_1$
8.334	0.39007	D_0(1v11)	$23b_2$
8.347	0.11205	D_0(1v13)	$22b_2$
8.365	0.14352	D_0(1v15)	$21b_2$
8.372	0.26407	D_0(1v14)	$6a_1$
8.384	0.87568	D_0(1v18)	$4a_1$
8.385	0.08828	D_0(1v16)	$5a_1$
8.419	0.14423	D_0(1v4,1v11)	$11a_2$,$23b_2$
8.457	0.09764	D_0(1v14,1v4)	$6a_1$,$11a_2$
8.468	0.07772	D_0(2v11)	$23b_2$
8.469	0.32378	D_0(1v4,1v18)	$23b_2$,$4a_1$
8.495	0.11204	D_0(1v8,1v18)	$24b_2$,$4a_1$
8.514	0.08363	D_0(1v10,1v18)	$8a_1$,$4a_1$
8.518	0.34158	D_0(1v11,1v18)	$23b_2$,$4a_1$
8.531	0.09813	D_0(1v13,1v18)	$22b_2$,$4a_1$
8.549	0.12568	D_0(1v15,1v18)	$21b_2$,$4a_1$
8.556	0.23125	D_0(1v14,1v18)	$11a_2$,$4a_1$
8.569	0.07731	D_0(1v16,1v18)	$5a_1$,$4a_1$
8.603	0.12630	D_0(1v4,1v11,1v18)	$11a_2$,$23b_2$,$4a_1$
8.641	0.08550	D_0(1v14,1v4,1v18)	$6a_1$,$11a_2$,$4a_1$

TABLE S17. Vibrational progressions in the RAES due to deexcitation from the intermediate core-excited state S_4 to the final ionised state D_0 calculated at the SRC1-R1/aug-cc-pVTZ level using the parallel normal modes approximation. The vibrational state assignment is provided as state(number of quanta—v—normal mode's number). Intensities were normalized to 1.

VALENCE PHOTOELECTRON SPECTROSCOPY

Trans.	EOM-IP-CCSD	EOM-IP-CCSD(fT)	ADC(3)	RASPT2(19/14)	RASPT2(19/14)
	aug-cc-pVTZ	u-6-311(2+,+)G**	aug-cc-pVTZ	aug-cc-pVTZ	ANO-L-TZVP
$9a_1$	13.12(0.9455)	12.98(0.9461)	13.19(0.8916)	12.84(0.8643)	12.86(0.8603)
$8a_1$	14.73(0.9446)	14.67(0.9452)	14.87(0.8873)	14.46(0.8455)	14.49(0.8423)
$7a_1$	18.09(0.9205)	18.03(0.9208)	17.63(0.2366),18.08(0.4404)	17.56(0.5979), 19.52(0.1281)	17.65(0.5141)
$6a_1$	19.41(0.9219)	19.32(0.9225)	-	18.90(0.4614)	19.05(0.3412), 19.53(0.2642)
$1a_2$	8.33(0.9489)	8.22(0.9490)	8.18(0.8876)	8.26(0.8602)	8.23(0.8599)
$6b_2$	13.57(0.9466)	13.53(0.9471)	13.62(0.8920)	13.27(0.8521)	13.31(0.8450)
$5b_2$	14.58(0.9441)	14.47(0.9446)	14.68(0.8820)	14.34(0.84475)	14.40(0.8423)
$4b_2$	18.65(0.9198)	18.57(0.9202)	18.37*	18.06(0.5658)	18.16(0.5468)
$2b_1$	9.18(0.9451)	8.98(0.9454)	9.13(0.8797)	9.08(0.8630)	9.05(0.8627)
$1b_1$	13.76(0.9096)	13.63(0.9085)	12.75(0.2157),14.22(0.5140)	12.87(0.5292),16.14(0.2855)	12.84(0.5289), 16.11(0.2851)

TABLE S18. Benchmark valence band ionisation energies of pyrrole in eV at various levels of theory. *not converged

VIBRATIONALLY RESOLVED VALENCE PHOTOELECTRON SPECTRUM

$S_0 \rightarrow D_0$		
Energy / eV	Intensity	Vibrational Transition
8.200	1.00000	D_0(0v0)
8.308	0.21213	D_0(1v8)
8.330	0.15982	D_0(1v10)
8.334	0.19712	D_0(1v11)
8.346	0.02913	D_0(1v13)
8.372	0.11474	D_0(1v14)
8.365	0.22448	D_0(1v15)
8.384	0.27067	D_0(1v16)
8.397	0.05413	D_0(1v19)
8.464	0.03148	D_0(1v10,1v11)
8.468	0.02030	D_0(2v11)
8.495	0.03589	D_0(1v10,1v15)
8.491	0.04413	D_0(1v11,1v15)
8.514	0.04325	D_0(1v10,1v16)
8.506	0.02265	D_0(1v11,1v14)
8.518	0.05325	D_0(1v11,1v16)

TABLE S19. Vibrationally resolved photoelectron spectrum of pyrrole in the D_0 state calculated at the SRC1-R1/aug-cc-pVTZ level. The parallel normal modes approximation was employed for the construction of the spectrum. The vibrational state assignment is provided as state(number of quanta—v—normal mode's number). Intensities > 0.01 are provided, the spectrum was normalized to 1.

[1] M. Neeb, J.-E. Rubensson, M. Biermann, and W. Eberhardt, Coherent excitation of vibrational wave functions observed in core hole decay spectra of O_2, N_2 and CO, Journal of Electron Spectroscopy and Related Phenomena **67**, 261 (1994).

[2] E. Pahl, H.-D. Meyer, and L. Cederbaum, Competition between excitation and electronic decay of short-lived molecular states, Zeitschrift für Physik D Atoms, Molecules and Clusters **38**, 215 (1996).

[3] G. Herzberg, *Molecular Spectra and Molecular Structure II: Infrared and Raman Spectra of Polyatomic Molecules* (Krieger, Malabar, 1991).
